# Mit1 Transcription Factor Mediates Methanol Signaling and Regulates the Alcohol Oxidase 1 (*AOX1*) Promoter in Pichia pastoris*[Fn FN2]

**DOI:** 10.1074/jbc.M115.692053

**Published:** 2016-01-31

**Authors:** Xiaolong Wang, Qi Wang, Jinjia Wang, Peng Bai, Lei Shi, Wei Shen, Mian Zhou, Xiangshan Zhou, Yuanxing Zhang, Menghao Cai

**Affiliations:** From the ‡ State Key Laboratory of Bioreactor Engineering, East China University of Science and Technology, Shanghai 200237, China and; the § Shanghai Collaborative Innovation Center for Biomanufacturing (SCICB), Shanghai 200237, China

**Keywords:** DNA-protein interaction, protein domain, signal transduction, transcription coactivator, transcription regulation, *AOX1* promoter, Mit1, *Pichia pastoris*, glycerol repression, methanol induction

## Abstract

The alcohol oxidase 1 (*AOX1*) promoter (P*_AOX1_*) of *Pichia pastoris* is the most powerful and commonly used promoter for driving protein expression. However, mechanisms regulating its transcriptional activity are unclear. Here, we identified a Zn(II)_2_Cys_6_-type methanol-induced transcription factor 1 (Mit1) and elucidated its roles in regulating P*_AOX1_* activity in response to glycerol and methanol. Mit1 regulated the expression of many genes involved in methanol utilization pathway, including *AOX1*, but did not participate in peroxisome proliferation and transportation of peroxisomal proteins during methanol metabolism. Structural analysis of Mit1 by performing domain deletions confirmed its specific and critical role in the strict repression of P*_AOX1_* in glycerol medium. Importantly, Mit1, Mxr1, and Prm1, which positively regulated P*_AOX1_* in response to methanol, were bound to P*_AOX1_* at different sites and did not interact with each other. However, these factors cooperatively activated P*_AOX1_* through a cascade. Mxr1 mainly functioned during carbon derepression, whereas Mit1 and Prm1 functioned during methanol induction, with Prm1 transmitting methanol signal to Mit1 by binding to the *MIT1* promoter (P*_MIT1_*), thus increasingly expressing Mit1 and subsequently activating P*_AOX1_*.

## Introduction

Methylotrophic yeasts such as *Candida boidinii*, *Hansenula polymorpha* (*Pichia angusta*), *Pichia methanolica*, and *Pichia pastoris* (*Komagataella pastoris*) are prominent hosts for producing heterologous proteins (1–5). The most important feature of methylotrophic yeasts is that most of the genes involved in the methanol utilization (MUT)^2^ pathway are repressed by glucose and are strongly induced by methanol (6). Promoters of genes involved in the MUT pathway, such as those encoding alcohol oxidase (*AOD1*, *MOX*, *MOD1*, or *AOX1* in the above species) and dihydroxyacetone synthase (*DAS* or *DHAS*), have been used to express heterologous proteins (7, 8). The *AOX1* promoter (P*_AOX1_*) of *P. pastoris* is one of the most widely used promoters for expressing large number of proteins, including antibodies, enzymes, cytokines, plasma proteins, and hormones (2). The activity of P*_AOX1_* is strongly repressed by multiple carbon sources such as glucose and glycerol, and is strongly induced by methanol (6, 9). This characteristic of P*_AOX1_* is extremely important for inducing the expression of heterologous proteins, especially toxic proteins (10).

Metabolic pathways and enzymes involved in the MUT pathway are similar in different methylotrophic yeasts (6, 11, 12). However, the gene transcriptional regulatory profiles are different. An important example is the gene for alcohol oxidase, such as *AOX1*in *P. pastoris*, *AOD1* in *C. boidinii*, *MOX* in *H. polymorpha*, and *MOD1* in *P. methanolica*. Although the promoters of these genes are repressed in the presence of glucose and ethanol, *AOD1* promoter (P*_AOD1_*) and *MOX* promoter (P*_MOX_*) show ∼3–30% and 60–80% derepression, respectively, whereas P*_AOX1_* shows almost complete repression in glycerol (6, 13). These differences cannot be attributed to variances in the sequences of these promoters because P*_AOX1_* has the same regulatory profile as P*_MOX_* when introduced into *H. polymorpha* (14). The differences in the derepression of various yeast promoters may be attributed to their unique *trans*-acting factors. However, these factors and their regulatory mechanisms are unclear.

Several transcription regulators are essential for expressing genes involved in the MUT pathway. These transcription regulators have been divided into three categories, namely (i) *H. polymorpha* Mpp1 (15), (ii) *P. pastoris* Mxr1 (16) and *C. boidinii* Trm2 (13), and (iii) *C. boidinii* Trm1 (17) and *P. pastoris* Prm1 (also called Trm1) (18, 19), based on their homology. Mpp1 regulates the expression of various proteins involved in peroxisome biogenesis (peroxins) and function (enzymes) in *H. polymorpha*. A study has shown that Δ*mpp1* cells has only one peroxisome and strongly decreases and completely represses the expression of peroxisomal matrix proteins Mox and Das, respectively (15). Mxr1 plays an important role in inducing the transcription of *AOX1* and other genes involved in the MUT pathway and that of *PEX* genes in *P. pastoris*. Mxr1 specifically binds to six sites within P*_AOX1_* (16, 20, 21). The function of Trm2 in *C. boidinii* is the same as that of Mxr1 in *P. pastoris* (13). Trm1, a methanol-specific gene activator, activates several methanol-inducible promoters in *C. boidinii* (17). Similarly, Prm1 functions as a positive regulator of genes involved in the MUT pathway in *P. pastoris* (18, 19). Thus, each of these transcription regulators plays a critical role in activating methanol-inducible promoters in methylotrophic yeasts. However, their interactions and synergistic functions have not yet been reported.

In this study, we identified methanol-induced transcription factor 1 (Mit1) in *P. pastoris* as an essential regulator of P*_AOX1_*. Although Mit1 has a Zn(II)_2_Cys_6_-type DNA-binding domain similar to *H. polymorpha* Mpp1, a BLAST homology search indicated that the complete amino acid (aa) sequence of Mit1 shows low identity to that of Mpp1 and other proteins with undetermined function. The present study explored the specific roles of Mit1 in the strict repression in glycerol medium and strong induction of P*_AOX1_* in methanol medium, respectively. More importantly, the study elucidates the regulatory profiles of Mit1 with other transcription activators in transduction of the methanol signal for activating P*_AOX1_*. The results of this study will help clarify in detail the mechanisms underlying the regulation of P*_AOX1_* in *P. pastoris* and will provide a reference for elucidating the mechanisms underlying the response of methylotrophic yeasts to methanol.

## Experimental Procedures

### 

#### 

##### Strains, Media, and Cultivation Conditions

The *P. pastoris* and *Escherichia coli* strains used in this study are listed in supplemental Table S1. *P. pastoris* GS115 (Invitrogen) was used as the wild type (WT) strain. *P. pastoris* strains were grown at 30 °C in YPD medium (1% yeast extract, 2% peptone, and 2% glucose) or minimal YNB medium (0.67% yeast nitrogen base without amino acids) supplemented with different carbon sources, *e.g.* 1% glucose (YND), 1% glycerol (YNG), 1% sorbitol (YNS), 0.5% methanol (YNM), 1% ethanol (YNE), or 0.5% oleate with 0.05% Tween 80 (YNO). Amino acids (50 μg ml^−1^) were used to support the growth of auxotrophic strains. For growth on plates, 2% agar powder was added. Electroporation was used for *P. pastoris*, and zeocin, hygromycin B, blasticidin S HCl, or G418 was added to a final concentration of 100, 750, 600, or 250 μg ml^−1^, respectively, for screening of transformants (22).

*E. coli* TOP10 and BL21(DE3) cells were used for plasmid propagation and the expression of recombinant proteins, respectively. *E. coli* strains were cultivated at 37 °C in LB medium (1% tryptone, 0.5% yeast extract, and 0.5% NaCl). When required, ampicillin, kanamycin, blasticidin S HCl, or zeocin was added to LB medium at a final concentration of 50–100 μg ml^−1^. Standard recombinant DNA techniques were adopted to construct plasmids. Plasmids and primers are listed in supplemental Tables S2 and S3, respectively.

##### Construction of Δmit1, Δprm1, and Δmxr1 Strains

The Δ*mit1*, Δ*prm1*, and Δ*mxr1* strains were generated by replacing their ORFs with hygromycin B or zeocin resistance gene (*hph* or *sh ble*) by a double crossover recombination. The Δ*mit1* strain was generated by replacing the ORF of *MIT1* with the hygromycin resistance gene (*hph*). First, the downstream region of the *MIT1* gene was amplified by PCR using genomic DNA as a template with *Pyrobest* DNA polymerase (TaKaRa). The primers MIT1Do5 and MIT1Do3 (supplemental Table S3) carry restriction sites for EcoRI and SalI. The resultant fragment was inserted into the EcoRI/SalI-digested plasmid pPIC3.5K (Invitrogen), yielding pMIT1Down. Second, the upstream region of *MIT1* and *hph* were amplified by PCR using primer pairs MIT1Up5/MIT1Up3 and Hyg5/Hyg3 (supplemental Table S3), respectively. These two fragments were ligated by overlapping PCR. Then the 2.4-kb connective fragment was inserted into SnaBI-digested plasmid pMIT1Down, yielding pMIT1-del. After pMIT1-del was digested with BamHI and SalI, the 4.1-kb fragment obtained was gel-purified and transformed into *P. pastoris* GS115. Transformants were isolated on YPD medium supplemented with 750 μg of ml^−1^ hygromycin B. The positive transformants were confirmed by PCR analysis and DNA sequencing.

The *PRM1* gene was disrupted by gene replacement using the zeocin resistance gene *Sh ble* as a marker. The deletion cassette was constructed as follows. First, two DNA fragments comprising regions −816 to −1 and +1,042 to +1,841 of the *PRM1* genomic region were obtained by PCR using primer pairs PRM1Up5/PRM1Up3 and PRM1Do5/PRM1Do3 (supplemental Table S3), respectively. The *Sh ble* gene was amplified from pGAPZB by PCR using primers Zeo5 and Zeo3 (supplemental Table S3). Subsequently, these three fragments and the pUC19 plasmid were digested by EcoRI + BamHI, SalI + SphI, BamHI +SalI, and EcoRI + SalI, respectively. Then the four digested fragments were ligated to yield the plasmid pPRM1-del. The deletion cassette was released from pPRM1-del by EcoRI + SalI and then transformed into *P. pastoris* GS115. Transformants were screened by using 100 μg ml^−1^ zeocin. The positive transformants were confirmed in the same way as the Δ*mit1* strain. The Δ*mxr1* strain was constructed in the same manner as the Δ*prm1* strain using the zeocin resistance gene *Sh ble* as a marker and MXR1Up5/MXR1Up3, MXR1Do5/MXR1Do3, and Zeo1/Zeo2 as primers.

The Δ*mit1*, Δ*prm1*, and Δ*mxr1* strains that were generated were subsequently confirmed by complementing *MIT1*, *PRM1*, and *MXR1* genes, respectively. *MIT1*, *PRM1*, and *MXR1* complementation strains were constructed as follows. First, the *MIT1* or *PRM1* gene with its corresponding 1000-bp upstream sequence was cloned by PCR with primer pairs BlnI-PMIT1–5/MIT1–3-SalI or SacI-PPRM1–5/PRM1–3-XhoI, resulting in fragment P*_MIT1_-MIT1* or P*_PRM1_-PRM1*, respectively. Then the P*_MIT1_-MIT1* fragment was digested by BlnI + SalI and subsequently inserted into the same sites of pGAPZB (Invitrogen), producing the complementation plasmid pGMM1 (supplemental Table S2). P*_PRM1_-PRM1* fragment was digested by SacI +XhoI. *AOX1* terminator (*AOX1*TT) fragment was amplified by XhoI-AOX1TT-5/AOX1TT-3-SpeI primers and digested by XhoI + SpeI. pAG32 was digested by SacI + SpeI. These three fragments were ligated, resulting in the complementation plasmid pAPP1 (supplemental Table S2). The *MXR1* gene with its 1000-bp upstream sequence was cloned by PCR with primer pairs PMXR1–5/MXR1–3, resulting in the P*_MXR1_-MXR1* fragment. pPGG (supplemental Table S2) was digested by BlnI to rescue the 7.7-kb fragment. Then these two fragments were ligated using a One Step Cloning Kit (Vazyme Biotech Co., Ltd.) to generate pPXX1 plasmid. Finally, pGMM1, pAPP1, and pPXX1 were digested by KpnI, AsuII, and XbaI and integrated into the P*_MIT1_*, P*_PRM1_*, and *his4* sites of the Δ*mit1*, Δ*prm1*, and Δ*mxr1* strains, respectively. The resulting strains were named Δ*mit1*-Mit1, Δ*prm1*-Prm1, and Δ*mxr1*-Mxr1, respectively (supplemental Table S1). Complementary strains Δ*mit1*-Mit1, Δ*prm1*-Prm1, and Δ*mxr1*-Mxr1 recovered their growth on methanol (data not shown).

##### Construction of Mpp1 Complementation Strain in P. pastoris

A 1000-bp upstream sequence of *MIT1* (P*_MIT1_* fragment) was cloned by PCR with BlnI-PMIT1–5/PMIT1-o3-Mpp1. The *MPP1* gene was amplified from the genome of *H. polymorpha* CBS4732 using MPP1-o5-PMIT1/MPP1–3-SalI primers (supplemental Table S3). P*_MIT1_* and *MPP1* fragments were ligated by overlapping PCR, resulting in the P*_MIT1_-MPP1* fragment. The fragment was then digested by *Bln*I + SalI and subsequently inserted into the same sites of pGAPZB (Invitrogen), resulting in the complementation plasmid pGM-Mpp1. It was then digested by KpnI and transformed into the Δ*mit1* mutant to construct the Δ*mit1*-Mpp1 strain.

##### Construction of GFP and BFP-SKL Expression Strains

To detect the strength of P*_AOX1_*, a plasmid pP-GFP (23) was linearized by SalI and transformed into the WT, Δ*mit1*, Δ*prm1*, Δ*mxr1*, and Δ*mit1*-Mpp1 strains, respectively. Strains containing a single copy of integrated plasmid were screened and designated WTA, Δ*mit1*A, Δ*prm1*A, Δ*mxr1*A, and Δ*mit1*A-Mpp1, respectively (supplemental Table S1). As a negative control, pPGG was constructed by replacing P*_AOX1_* of pP-GFP with the *GAP* promoter (P*_GAP_*) (supplemental Table S2). pPGG was then linearized by SalI and transformed into WT, Δ*mit1*, and Δ*prm1* cells, respectively. Strains containing a single copy of integrated plasmid were screened and designated WTG, Δ*mit1*G, and Δ*prm1*G, respectively (supplemental Table S1).

To measure the regulatory profiles of P*_MIT1_* and the *PRM1* promoter (P*_PRM1_*) by different carbon sources, plasmids named pGMG and pAPG containing P*_MIT1_*-GFP-*AOX1*TT and P*_PRM1_*-GFP-*AOX1*TT expression cassettes were constructed (supplemental Table S2). Firstly, the P*_MIT1_*, P*_PRM1_*, and GFP coding fragments were amplified and ligated by overlapping PCR to yield P*_MIT1_*-GFP and P*_PRM1_*-GFP fragments, respectively. P*_MIT1_*-GFP and P*_PRM1_*-GFP fragments were subsequently digested with *Bln*I + XhoI and SacI + XhoI and then inserted into the corresponding restriction sites of pGAPZB (Invitrogen) and pAPP1 to produce pGMG and pAPG, respectively. pGMG and pAPG were then linearized by KpnI and AsuII, respectively, and then transformed into the strains of Δ*mit1* or Δ*prm1* and WT. Strains containing a single copy of P*_MIT1_*-GFP-*AOX1*TT or P*_PRM1_*-GFP-*AOX1*TT cassette were selected and named WT-P*_MIT1_*-GFP, WT-P*_PRM1_*-GFP, Δ*mit1*-P*_MIT1_*-GFP, and Δ*prm1*-P*_PRM1_*-GFP, respectively (supplemental Table S1).

To visualize the peroxisome morphology of the Δ*mit1* or Δ*prm1* strain, we constructed the plasmid pPWBS wherein the BFP-SKL fusion protein is under the control of a strong constitutive *GCW14* promoter (P*_GCW14_*) (24). First, the P*_GCW14_* promoter was amplified by PCR using SacI-PGCW14–5/PGCW14–3-EcoRI with the *P. pastoris* genome as template. The blue fluorescent protein (BFP) fragment was amplified from pRDM054 using EcoRI-BFP-5/BFP-3-NotI primers (supplemental Table S3). These two fragments was digested by SacI +EcoRI and EcoRI + NotI, respectively, and inserted into the SacI/NotI sites of pP-GFP. pPWBS was then digested by SalI and transformed into WT, Δ*mit1*, and Δ*prm1* to construct the WT-BFP-SKL, Δ*mit1*-BFP-SKL, and Δ*prm1*-BFP-SKL strains (supplemental Table S1).

##### Construction of Domain Deletion Mutants

Domain deletion fragments of Mit1 fusing with FLAG tag at their C terminus were generated by overlapping PCR using mutagenic primers and flanking primers (supplemental Table S3) and then inserted into KpnI/SalI sites of pGMM1 plasmid, resulting in a series of domain deletion plasmids (supplemental Table S2). These domain deletion plasmids of Mit1 were linearized by KpnI and subsequently integrated into the Δ*mit1*A genome from the P*_MIT1_* region by electroporation to produce the desired strains (supplemental Table S1). The stable expression of these Mit1 mutants was detected by Western blotting using anti-FLAG antibody.

##### Electrophoretic Mobility Shift Assays (EMSA)

*P. pastoris* Mit1 (aa 1–150) and Prm1 (aa 41–90) were expressed in *E. coli* and purified by Ni^2+^-NTA His bind resin. Cy5-labeled fragments of P*_AOX1_* (−940 to −704, −723 to −515, −534 to −367, and −386 to −162 bp of *AOX1*), P*_MIT1_* (−1,000 to −701, −800 to −601, −700 to −351, −450 to −251, and −350 to −1 bp of *MIT1*), or P*_PRM1_* (−699 to −540, −562 to −314, and −338 to −112 bp of *PRM1*) were incubated with the zinc finger domains of *P. pastoris* Mit1 (aa 1–150) and/or Prm1 (aa 41–90) in a 20-μl reaction containing binding buffer for 30 min at 4 °C. The binding buffer contained 50 mm Tris-HCl (pH 8.0), 50 mm NaCl, 1 mm DTT, 0.05% Nonidet P40, 50 μg·ml^−1^ poly[d(I-C)], and 5% glycerol. In some experiments, an unlabeled specific fragment (200-fold) or nonspecific competitor DNA (200-fold, sonicated salmon sperm DNA) was used and incubated with proteins for 5 min prior to the addition of Cy5-labeled fragments. Samples were loaded onto native 6% polyacrylamide gels and electrophoresed in 0.5× TBE (45 mm Tris, 45 mm boric acid, and 1 mm EDTA (pH 8.3)). The DNA fragments or fragment shifts were visualized by measuring the fluorescence signal using the Typhoon Trio system (GE Healthcare Life Sciences).

##### DNase I footprinting Assay

A DNase I footprinting assay was performed according to Zianni *et al*. (25). For preparation of fluorescent 6-carboxyfluorescein (FAM)-labeled probes, the promoter fragments of P*_AOX1_* were amplified from the plasmid pMD19T inserted with specific promoter fragments of P*_AOX1_* using primers M13F-47 (FAM-labeled) and M13R-48 (supplemental Table S3). The FAM-labeled probes were purified using Wizard® SV Gel and the PCR Clean-up System (Promega) and were quantified with NanoDrop 2000c (Thermo Scientific). For each assay, 350-ng probes were incubated with different amounts of Mit1 (aa 1–150) or Prm1 (aa 41–90) in a total volume of 40 μl. After incubation for 30 min at 25 °C, 10 μl of solution containing about 0.015 unit of DNase I (Promega) and 100 nmol of freshly prepared CaCl_2_ was added and further incubated for 1 min at 25 °C. The reaction was stopped by adding 140 μl of DNase I stop solution (200 mm unbuffered sodium acetate, 30 mm EDTA, and 0.15% SDS). Samples were first extracted with phenol/chloroform and then precipitated with ethanol, and the pellets were dissolved in 30 μl of MiniQ water. The preparation of the DNA ladder, electrophoresis, and data analysis were the same as described previously (25), except that the GeneScan-500 LIZ size standard (Applied Biosystems) was used.

##### Chromatin Immunoprecipitation Assay

A chromatin immunoprecipitation (ChIP) assay was performed in Δ*mit1*-Mit1-FLAG and Δ*prm1*-Prm1-HA strains (supplemental Table S1), wherein FLAG and HA were expressed at the C terminus of Mit1 and Prm1 under the control of their native promoters, respectively. Δ*mit1*-Mit1-FLAG and Δ*prm1*-Prm1-HA were cultivated in glucose, glycerol, or methanol for 3 h, respectively. Whole cell proteins were extracted and subsequently used for the ChIP assay. The ChIP assay was performed as described previously (26) except that immunoprecipitation was performed by anti-FLAG (Cell Signaling, 1:200 dilution, catalog No. 14793, lot 4) or anti-HA (Abcam, 1:200 dilution, catalog No. ab18181, lot GR14161-4) monoclonal antibody, respectively. The specific primer pairs PACT1-F/PACT1-R for *ACT1* promoter (P*_ACT1_*) and A1-F/A1-R, A2-F/A2-R, A3-F/A3-R, A4-F/A4-R, A5-F/A5-R, A6-F/A6-R, A7-F/A7-R, A8-F/A8-R, and A9-F/A9-R, for P*_AOX1_* are listed in supplemental Table S3. Occupancy of a protein is expressed as -fold increase of the immunoprecipitation to input ratio of the amount of the specific amplicon for the gene sequence over the immunoprecipitation to input ratio corresponding to the amplicon for P*_ACT1_*.

##### Construction of Strains Expressing GFP-Mit1 or GFP-Prm1 Fusion Protein

Strains expressing GFP-Mit1 or GFP-Prm1 fusion protein were constructed to visualize the localization of Mit1 or Prm1 by fluorescence microscopy, respectively. First, the *MIT1* or *PRM1* coding region was amplified by PCR using genomic DNA as a template with the primer pairs NotI-LMIT1–5/MIT1–3-SalI or NotI-LPRM1–5/PRM1–3-SalI carrying restriction sites for NotI and SalI. Subsequently, the resulted fragment of the amplified *MIT1* or *PRM1* coding region was digested by NotI + SalI, yielding a 2.6- or 2.7-kb fragment. The fragments were ligated with a GFP fragment by a shot linker peptide (GGGRS) and then inserted into pGAPZB, yielding pGGLMit1 or pGGLPrm1, respectively (supplemental Table S2). pGGLMit1 or pGGLPrm1 was then linearized with BlnI and transformed into the GS115 strain. Transformants containing zeocin resistance were isolated. The resulting strains were named WT-GMit1 and WT-GPrm1, respectively (supplemental Table S1).

##### Construction of Strains for Methanol Induction Signal Transduction Analysis

To elucidate how methanol induction signal transmits in Mit1, Prm1, and Mxr1, strains of Δ*mit1*-Mxr1, Δ*mit1*-Prm1, Δ*prm1*-Mxr1, Δ*prm1*-Mit1, Δ*mxr1*-Mit1, and Δ*mxr1*-Prm1 overexpressing HA-tagged Mit1, Mxr1, or Prm1 were constructed. First, pPIC6A (Invitrogen) was digested by BglII + XhoI and then ligated with P*_GAP_* and fragment *MXR1*-HA using the One Step Cloning Kit (Vazyme) to obtain the plasmid pP6GX1. Second, pPIC3.5K (Invitrogen) was digested by ScaI + NotI and then ligated with P*_GAP_* and fragment *PRM1*-HA using the One Step Cloning Kit to obtain the plasmid pPGP1. Third, P*_GAP_*, *MIT1*-HA, and *AOX1* terminator fragments were ligated and then inserted into the SacI and SpeI sites of pAG32 to obtain the plasmid pAGM1. Finally, pP6GX1, pPGP1, and pAGM1 were linearized by BlnI and transformed to the Δ*mit1*A, Δ*mxr1*A, and Δ*prm1*A mutants to construct strains of Δ*mit1*-Mxr1, Δ*prm1*-Mxr1, Δ*mit1*-Prm1, Δ*mxr1*-Prm1, Δ*prm1*-Mit1, and Δ*mxr1*-Mit1, respectively. The stable expression of Mxr1-HA, Mit1-HA, and Prm1-HA was detected by Western blotting using anti-HA antibody.

##### Ni^2+^-NTA Pulldown Assay

To perform the Ni^2+^-NTA pulldown assay, the WT-PXM, WT-PM, and WT-XB strains were constructed, wherein Prm1-HA, Mxr1-His_6_, and Mit1-FLAG, Prm1-HA and Mit1-His_6_, or Mxr1-His_6_ and Bmh1-FLAG fusion proteins were expressed under the control of their native promoters or P*_GAP_*, respectively (supplemental Table S1). The WT-XB strain was used as a positive control for analysis of the interaction between Mxr1-His_6_ and Bmh1-FLAG. The WT-PXM strain was used to detect the interactions between Mxr1-His_6_ and Mit1-FLAG (or Prm1-HA), and the WT-PM strain was used to detect the interactions between Mit1-His_6_ and Prm1-HA.

Yeast cells were cultivated in methanol. Then whole cell extract containing ∼10 mg (2 ml) of total protein was incubated with Ni^2+^-NTA beads at 4 °C for 1 h with continuous rotation in binding buffer (50 mm Tris-HCl (pH 8.0), 200 mm NaCl, and 5% glycerol). The beads were then pelleted by centrifugation at 500 × *g* for 1 min, washed three times with washing buffer (binding buffer + 20 mm imidazole), and resuspended in 20 μl of 2× SDS-PAGE loading buffer followed by boiling for 10 min. Three independent replicates were performed and the pulled-down proteins were mixed together. Then, 20 μg of total whole cell extract or 20 μl of pulled-down proteins were loaded into each lane for Western blotting using antibodies of their specific tags.

##### Bacterial Two-hybrid (B2H) Assay

Protein-protein interactions were detected using the Euromedex bacterial two-hybrid (BACTH) system as per the manufacturer's manual. Two candidate proteins were fused to pUT18C and pKT25 (supplemental Table S2) using the One Step Cloning Kit (Vazyme). Two complementary plasmids were transformed into *E. coli BTH101*, and transformants that could grow on LB plates supplemented with ampicillin and kanamycin were isolated. Strains with proteins that could bind with each other showed up as blue colonies on LB plates supplemented with isopropyl 1-thio-β-d-galactopyranoside and β-galactosidase. Strains, plasmids, and primers used in the yeast two-hybrid assay are listed in supplemental Tables S1, S2, and S3, respectively.

##### Bimolecular Fluorescence Complementation (BiFC) Assay

A BiFC assay was performed as follows. First, the N-terminal aa 1–173 of YFP protein and one candidate protein were fused with the linker peptide RSIAT (27). The fused protein was then inserted to multiple cloning sites of pGAPZA. Then the C-terminal aa 155–239 of YFP protein and the other candidate protein were fused with the linker peptide RPACKIPNDLKQKVMNH (27). The fused protein was then inserted to pAGM1 (supplemental Table S2) to replace the *MIT1* fragment. The two resulting plasmids were linearized by BlnI and then co-transformed into wild type cells. Positive interactions resulted in yellow fluorescence. Strains, plasmids, and primers used in the BiFC assay are listed in supplemental Tables S1, S2, and S3, respectively.

##### Miscellaneous Methods

BFP and GFP were visualized by inverted microscope DMI3000B (Leica) using a ×100 oil immersion objective. Images were processed using the Leica application suite, version 2.8.1. Cell-free extract preparations, enzyme assays, fluorescence microscopy, quantitative real-time reverse transcription-PCR, and Western blotting were adapted from Zhang *et al.* (9). For Western blotting assays, 20 μg of total protein (measured by the Bradford protein assay kit (TianGen Biotech)) were loaded into each gel lane. For immunoprecipitated proteins, 20 μl of the same pulled-down protein sample was loaded into each gel lane. The gray levels of the Western blots were analyzed by Gel-Pro Analyzer 4 and then normalized to total protein (also verified by SDS-PAGE; data not shown) to ensure experimental accuracy. Specific primers used for quantitative real-time reverse transcription-PCR are listed in supplemental Table S2. For Western blotting, rabbit anti-FLAG antibody (Cell Signaling, 1:1,000 dilution, catalog No. 14793, lot 4), mouse anti-HA antibody (Abcam, 1:1,000 dilution, catalog No. ab18181, lot GR14161-4), mouse anti-His_6_ antibody (TianGen Biotech, 1:1,000 dilution, catalog No. AB102, lot M2112), or rabbit anti-beta actin antibody (Abcam, 1:1,000 dilution, catalog No. ab8224, lot GR14272-6) was used as the primary antibody, and peroxidase-conjugated goat anti-rabbit IgG (Beyotime Institute of Biotechnology, Jiangsu, China, 1:1,000 dilution, catalog No. A0208) or peroxidase-conjugated goat anti-mouse IgG (Beyotime Institute of Biotechnology, 1:1,000 dilution, catalog No. A0216) was used as the secondary antibody, respectively.

##### Statistical Analysis

All data were obtained from three biological replicates (each with three technical replicates) assayed in duplicate and presented as mean ± S.D. Student's *t* test was performed to determine the differences among grouped data. Statistical significance was assessed at *p* < 0.05 and *p* < 0.01.

## Results

### 

#### 

##### Identification of an Essential Transcription Factor Mit1 for the Growth of P. pastoris in Methanol

**FIGURE 1.**
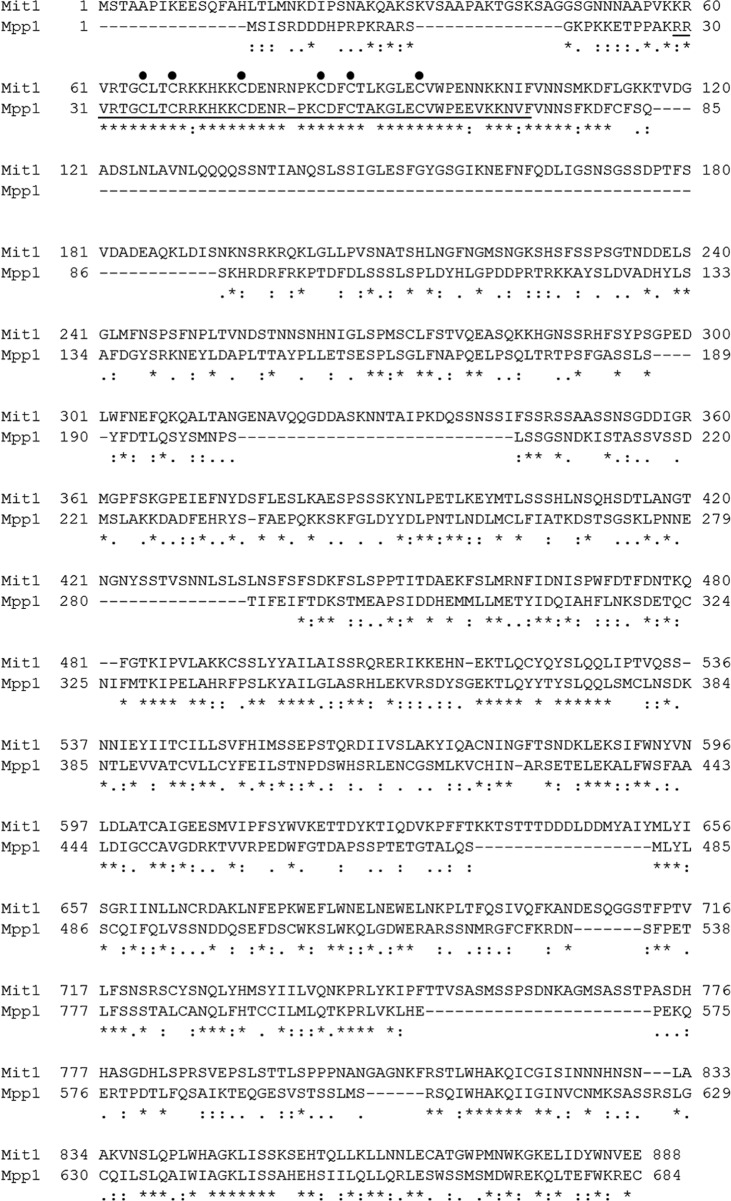
**Alignment of amino sequences of *P. pastoris* Mit1 (GenBank^TM^ accession number CAY70887) and *H. polymorpha* Mpp1 (GenBank^TM^ accession number AAO72735) by the ClustalX program.** The amino acids are shown by one-letter codes. Gaps were introduced to maximize the similarity. The zinc finger domain between aa 29 and 73 in Mpp1 is *underlined*. The *solid black circles* represent cysteine residues of Zn(II)_2_Cys_6_ domain. A *line below* the alignment was used to mark highly conserved positions. Three characters (*asterisk*, *colon*, and *period*) were used: *asterisk*, indicates positions that have a single, fully conserved residue; *colon*, indicates positions that are fully conserved in the “strong” groups; *period*, indicates positions that are fully conserved in the “weak” groups. The strong and weak groups were determined by ClustalX.

In our previous study, we identified a methanol-induced zinc finger (ZF) protein by performing RNA-Seq (data not published) and designated it as *P. pastoris* Mit1 (methanol-induced transcription factor; GenBank^TM^ accession number CAY70887). BLAST homology search indicated that Mit1 was a homolog of *H. polymorpha* Mpp1 (GenBank^TM^ accession number AAO72735), a novel transcription regulator of genes encoding peroxisomal proteins, especially *MOX* and *DHAS* (or *DAS*) in the presence of methanol (15). The N terminus of Mit1 has a Zn(II)_2_Cys_6_ -type DNA-binding domain (aa 65–93) that shows 85% identity and 91% similarity to the ZF domain of Mpp1, and its large C terminus (aa 343–887) shows 33% identity and 50% similarity to the C terminus (aa 203–683) of Mpp1 (Fig. 1). However, the results of a BLAST homology search indicated that the complete sequence of Mit1 shows low identity to that of Mpp1 and has few references to proteins of other microorganisms.

To elucidate the function of Mit1, we constructed a *MIT1*-deficient (Δ*mit1*) strain and measured its growth in the presence of glucose, glycerol, methanol, ethanol, sorbitol, or oleate as the sole carbon source. As shown in Table 1, Δ*mit1* cells could not grow in the presence of methanol; however, their generation time was unaffected in the presence of glucose, glycerol, ethanol, sorbitol, or oleate. This result indicates that Mit1 is essential for the growth of *P. pastoris* in methanol.

**TABLE 1:** **Generation times for Δ*mit1* and Δ*prm1* mutants grown on selected carbon sources** The mediums were minimal YNB medium (0.67% yeast nitrogen base without amino acids) and 50 mg ml^−1^ histidine supplemented with 1% glucose (YND), 1% glycerol (YNG), 1% sorbitol (YNS), 0.5% methanol (YNM), 1% ethanol (YNE), or 0.5% oleate with 0.05% Tween 80 (YNO).

Strain	Generation time					
	Glucose (YND)	Glycerol (YNG)	Methanol (YNM)	Ethanol (YNE)	Sorbitol (YNS)	Oleate (YNO)
				*h*:*min*		
WT	2:37	2:35	4:30	3:30	4:53	6:58
Δ*mit1*	2:43	3:11	No growth	3:40	5:15	7:08
Δ*prm1*	2:50	3:04	No growth	3:35	5:14	6:58

##### Mit1 Activates Genes Involved in the MUT Pathway but Does Not Participate in Peroxisome Proliferation

The catabolism of methanol depends on the genes involved in the MUT pathway as well as on the function of the peroxisomes (28). The transcription levels of the genes involved in the MUT pathway and peroxisome biogenesis were measured in Δ*mit1* cells exposed to methanol by performing quantitative reverse transcription-PCR. In addition, WT and Δ*prm1* cells were analyzed.

**FIGURE 2.**
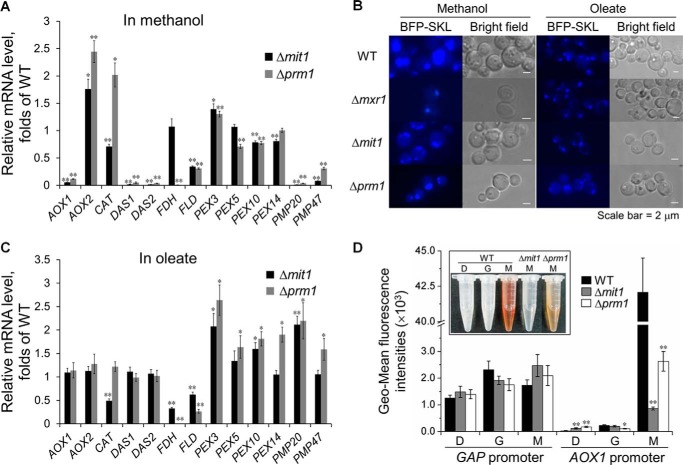
**Mit1 activates methanol utilization genes but keeps away from peroxisome functions.** *A* and *C*, transcription levels of genes involved in methanol utilization pathway and peroxisome biogenesis of the Δ*mit1* and Δ*prm1* strains grown in methanol (*A*) or oleate (*C*). The mRNA levels were normalized to the housekeeping gene *ACT1* in each sample. The relative expression level indicated on the *y* axis (2^−ΔΔCT^) for each gene was normalized to that in the WT cells grown in the corresponding carbon source. The *error bars* represent the standard deviation of three biological replicates, each with three technical replicates, assayed in duplicate. *B*, fluorescence microscopy images of Δ*mit1*, Δ*prm1*, Δ*mxr1*, and WT strains expressing the peroxisome-targeted BFP-SKL fusion protein in methanol and oleate. SKL was fused to the C terminus of BFP to localize BFP to the peroxisomes. The Δ*mit1*, Δ*prm1*, Δ*mxr1*, and WT cells were pregrown in YPD to log phase and washed three times in sterile water. The washed cell pellets were transferred to YNM medium (YNB + 0.5% methanol) and YNO medium (YNB + 0.5% oleate + 0.05% Tween 80) supplemented with the requisite amino acid. After culture at 30 °C for 1 h, aliquots of cell pellets were harvested and subsequently washed to visualize BFP-SKL. *D*, evaluation of the activity of P*_AOX1_* by colorable reaction of Aox and exploiting a reporter gene (*GFP*) expression assay in the Δ*mit1*, Δ*prm1*, and WT strains grown in glucose, glycerol, and methanol, respectively. For the GFP expression assay, the Δ*prm1* strain was used as a positive control. GFP expressed under the control of P*_GAP_* was used as a negative control. GFP expression in *P. pastoris* was analyzed using an enzyme-labeled instrument (BioTek Instruments) at an excitation wavelength of 488 nm and an emission wavelength of 512 nm. The fluorescence values were determined by a geometric mean (Geo-Mean) method. The *error bars* represent the standard deviation of three biological replicates, each with three technical replicates, assayed in duplicate. An independent-sample *t* test was used to determine the statistical significance of the mutant groups relative to the WT groups in the corresponding carbon sources and promoter. *, *p* < 0.05; ** *p* < 0.01. Colorable reaction was performed as per Zhang *et al.* (9). It was visualized by adding the Aox reaction mixture with the permeabilizing agent to the cell pellets for 30 min. The higher Aox activity corresponds to the *deeper red* of the reaction mixture. *D*, glucose; *G*, glycerol; *M*, methanol.

Exposure to methanol decreased the transcription of genes involved in the MUT pathway and genes of peroxisomal membrane proteins, and it even inhibited the transcription of *AOX1*, *DAS1*, *DAS2*, and *PMP20* in Δ*mit1* and Δ*prm1* cells compared with WT cells (Fig. 2*A*). In contrast to the deletion of *PRM1*, the deletion of *MIT1* did not affect the transcription of *FDH* (encoding formate dehydrogenase) (Fig. 2*A*). Next, we expressed BFP fused with SKL, a peroxisome-tagging sequence, under the control of P*_GCW14_*, a strong constitutive promoter (24), in all three cell types and examined these cells under a fluorescence microscope. We observed that peroxisomes of the Δ*mit1* and Δ*prm1* cells exposed to methanol were smaller than those of WT cells exposed to methanol; however, the proliferation of peroxisomes seemed normal (Fig. 2*B*). Abnormal or small peroxisomes are produced in cells lacking peroxisomal matrix proteins such as Aox and Das (17). This may be the reason for the formation of small peroxisomes in Δ*mit1* and Δ*prm1* cells. Aox and Das deficiency may also be responsible for the decreased mRNA expression of *PMP20* and *PMP47* (Fig. 2*A*).

To exclude the effect of decreased Aox and Das levels on peroxisomes, we measured the expression of genes encoding peroxisomal matrix proteins in the presence of oleate, because the metabolism of fatty acids occurs in peroxisomes and depends strictly on peroxisome function. The transcription of genes encoding Aox and Das was unaffected in Δ*mit1* and Δ*prm1* cells exposed to oleate (Fig. 2*C*). Moreover, the transcription of *PMP20* and *PMP47* did not decrease in these cells. In addition, we observed that Δ*mit1* and Δ*prm1* cells exposed to oleate had more than one peroxisome of a small size, similar to that found in WT cells (Fig. 2*B*). However, Δ*mxr1* cells exposed to oleate or methanol showed an obvious decrease in peroxisome proliferation, consistent with the result of a study showing growth defects in Δ*mxr1* cells exposed to oleate and methanol (16). Therefore, we suggest that Mit1 and Prm1 are mainly involved in the MUT pathway but do not participate in peroxisome proliferation and transportation of peroxisomal proteins.

The regulation of P*_AOX1_* generally occurs at the transcriptional level (7). To elucidate the mechanisms underlying the regulation of P*_AOX1_* by Mit1, we measured the expression of GFP or Aox under the control of P*_AOX1_*. The mRNA level of *AOX1* decreased dramatically in the absence of Mit1 (Fig. 2*A*). Moreover, the expression of GFP under the control of P*_AOX1_* in Δ*mit1* cells exposed to methanol was only 2.0% of that in WT cells exposed to methanol. A colorable reaction of Aox also accorded with the GFP expression level (Fig. 2*D*). These results indicated that Mit1 regulates P*_AOX1_* activity mainly at the transcriptional level and is essential for activating P*_AOX1_*. Similar results were obtained using Δ*prm1* cells (Fig. 2*D*), and these results were consistent with those reported in a previous study (19). However, the decreased expression of GFP and Aox under the control of P*_AOX1_* suggested that P*_AOX1_* activity is regulated more strictly by Mit1 than by Prm1 (Fig. 2*D*).

##### Mit1 Strictly Represses P_AOX1_ in the Presence of Glycerol

**FIGURE 3.**
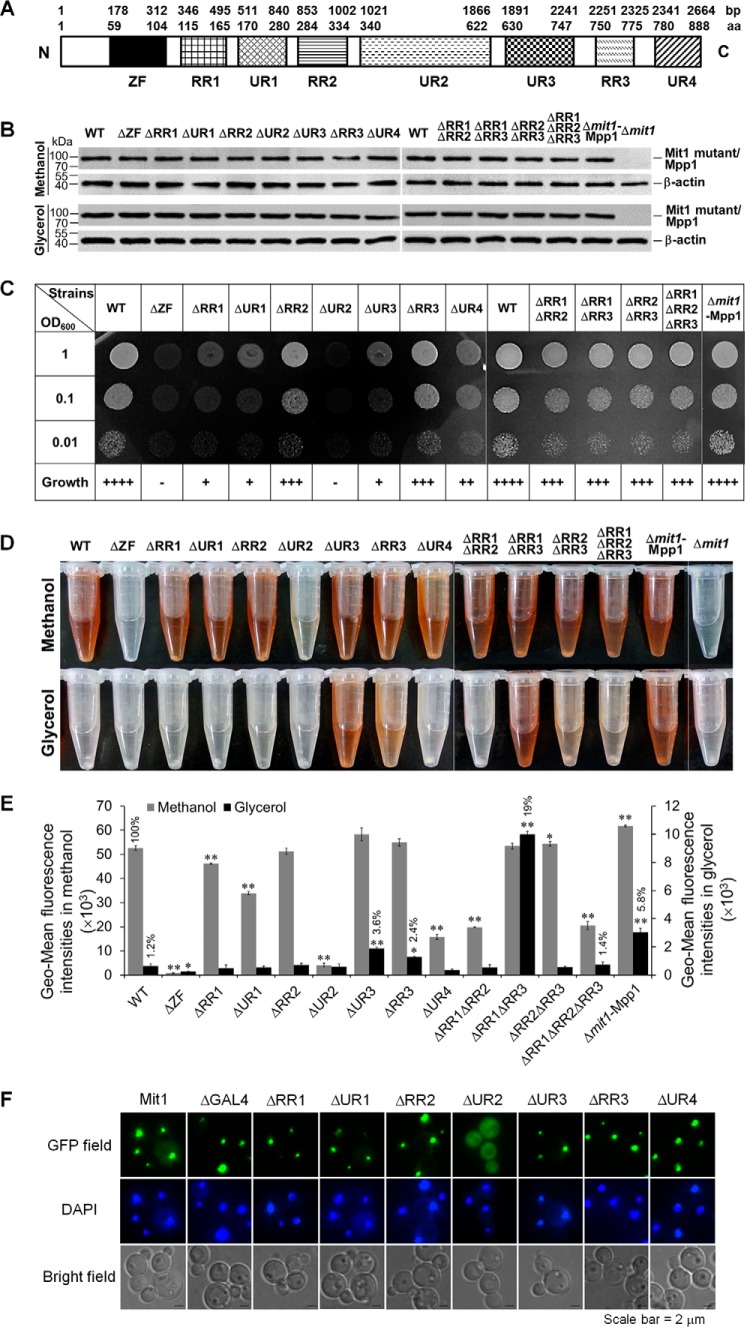
**Complementation of the *P. pastoris* Δ*mit1* mutant by *H. polymorpha* Mpp1 and domain-deleted Mit1.** *A*, schematic drawing of the annotated domains within Mit1. *ZF*, the GAL4-like Zn(II)_2_Cys_6_ (or C_6_ zinc) binuclear cluster DNA-binding domain. *B*, expression of Mpp1 and domain-deleted Mit1 in complementary cells in glycerol and methanol. Mpp1 and domain-deleted Mit1 were FLAG-tagged at the C terminus and detected by Western blotting using anti-FLAG antibody. Twenty micrograms of total protein was loaded into each lane, and the expression of β-actin was used as a positive control. Normalization of the signal intensity to total protein loading is described under “Miscellaneous Methods.” *C*, growth of Δ*mit1*-Mpp1 and domain deletion mutants of Mit1 in methanol. Cells were pregrown in YPD medium to log phase and washed three times in sterile water. The washed cell pellets were then diluted to an OD_600_ of 0.01, 0.1, and 1 (as indicated). Ten μl of each was spotted onto YNB plates containing the 0.5% methanol and the requisite histidine. Then the plates were incubated at 30 °C for about 3 days. ++++, the same growth as WT; +++, little growth defect compared with WT; ++, weak growth; +, little growth; −, no growth. *D*, colorable reaction of Aox in Δ*mit1*-Mpp1 and domain deletion mutants of Mit1 in methanol and glycerol. A colorable reaction was performed the same as described for Fig. 2. *E*, evaluation of the activity of P*_AOX1_* by exploiting a reporter gene (*GFP*) expression assay in Δ*mit1*-Mpp1, domain deletion mutants of Mit1, and the WT grown in methanol and glycerol. The GFP expression was measured the same as described for Fig. 2. Δ, strains with domain-deleted Mit1. The *error bars* represent the standard deviation of three biological replicates, each with three technical replicates, assayed in duplicate. An independent sample *t* test was used to determine the statistical significance of the mutant groups relative to WT groups in corresponding carbon sources. *, *p* < 0.05; **, *p* < 0.01. *F*, subcellular localization of Mit1 and domain-deleted Mit1 with GFP fusing at their N termini in methanol media. DAPI was used to stain the cell nucleus.

*H. polymorpha* P*_MOX_* showed ∼60–80% derepression, whereas *P. pastoris* P*_AOX1_* showed complete repression in the presence of glycerol (6, 13). Moreover, P*_AOX1_* introduced into *H. polymorpha* showed the same regulatory profile as P*_MOX_* (14), indicating that the difference in their regulation was due to their *trans-*acting factors and not their *cis*-acting elements. The low identity between Mit1 and Mpp1 (Fig. 1) may be one of the major reasons for this difference. Therefore, we analyzed the functional domains of Mit1 (Fig. 3*A*). GeneDoc alignment showed that the ZF domain of Mit1 was highly conserved and that Mit1 had three redundant regions, designated redundant regions 1, 2, and 3 (RR1, RR2, and RR3). Further, GeneDoc alignment predicted that Mit1 had four undefined regions, namely UR1, UR2, UR3, and UR4, that showed low homology to those in Mpp1.

**FIGURE 4.**
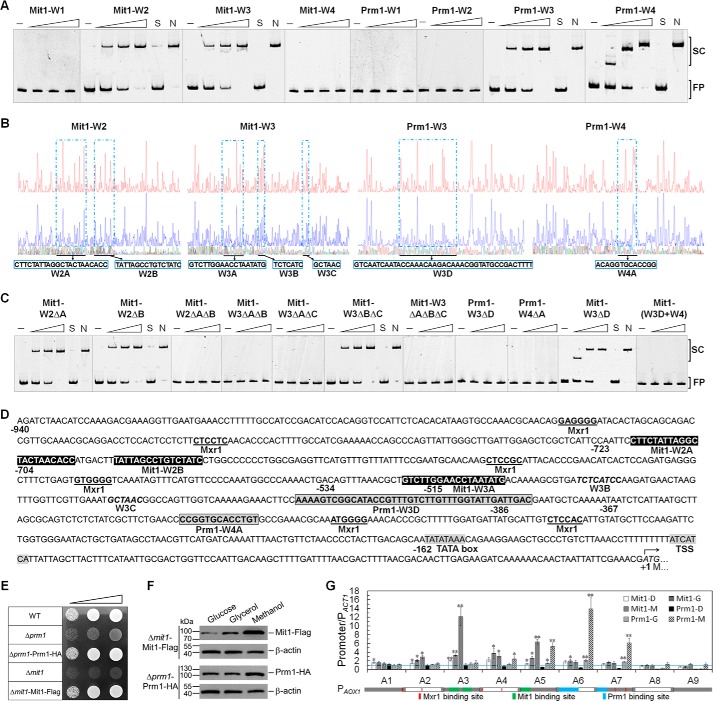
**Both Mit1 and Prm1 bind to *AOX1* promoter *in vivo* and *vitro*.** *A*, detection of binding characteristics of Mit1 and Prm1 to P*_AOX1_* fragments. EMSAs were performed by incubating Cy5-labeled fragments W1 (−940 to −704 bp), W2 (−723 to −515 bp), W3 (−534 to −367 bp), and W4 (−386 to −162 bp) of P*_AOX1_* with ZF domains of *P. pastoris* Mit1 (aa 1–150) and Prm1 (aa 41–90) expressed by *E. coli*. For each EMSA, about a 30 nm Cy5-labeled fragment and 0 (indicated by a *solid line*), 0.2, 0.5, and 1 μm (amount increase indicated by a *right-pointing triangle*) recombinant Mit1 or Prm1 were added. EMSAs with a 200-fold excess of unlabeled specific fragments (*S*) or nonspecific competitor fragment (sperm DNA) (*N*) were conducted as controls. The shifted protein-DNA complexes (*SC*) and free DNA probes (*FP*) are indicated on the *right. B*, electropherograms of DNase I digest of FAM-labeled W2, W3, and W4 fragments of P*_AOX1_* incubated without proteins (*top* of each *panel*) or with 4 μg of Mit1 (aa 1–150) (Mit1-W2 and Mit1-W3), 7.5 μg (Prm1-W3), or 1.4 μg (Prm1-W4) of Prm1 (aa 41–90) (*middle* of *each panel*), respectively. The respective nucleotide sequences bound by Mit1 (aa 1–150) and Prm1 (aa 41–90) are indicated (*bottom* of *each panel*). *C*, detection binding characteristics of Mit1 and Prm1 to mutant P*_AOX1_* fragments. EMSAs were performed by incubating Cy5-labeled fragments W2ΔA, W2ΔB, W2ΔAΔB, W3ΔAΔB, W3ΔAΔC, W3ΔBΔC, W3ΔAΔBΔC, W3ΔD, W4ΔA, and W3D + W4 with zinc finger domains of *P. pastoris* Mit1 (aa 1–150) or Prm1 (aa 41–90) expressed by *E. coli*. All other *labeling* is the same as in *A. D*, binding sites of Mit1, Prm1, and Mxr1 in the promoter regions of *AOX1*. The binding sites identified by both DNase I footprinting assays and EMSAs are in *bold*; specifically the Mxr1 binding sites are *underlined*, the Mit1 and Prm1 binding sites are shown within a *black shaded* and a *gray shaded rectangle*, respectively. The binding sites of Mit1 and Prm1 identified by DNase I footprinting assays but not by EMSAs are indicated with *italics*. The TATA box and transcriptional starting site (*TSS*) are indicated with a *light shadow*. The translation starting codon is indicated with *italics* and a *bent arrow*. Specific sites relative to the translational start site are indicated under the corresponding nucleotides. The *numbering* is relative to the translational start site (+1). The specific fragments or protein-fragment binding partners were indicated under the corresponding regions. *E*, growth of Δ*mit1*-Mit1-FLAG and Δ*prm1*-Prm1-HA cells in methanol media. Cells were pregrown in YPD medium to log phase and washed three times by sterile water. The washed cell pellets were then diluted to an OD_600_ of 0.01, 0.1, and 1 (as indicated by the *inclined triangle*). Then 10 μl of each was spotted onto YNB plates containing 0.5% methanol and the requisite amino acid histidine. *F*, expression of Mit1-FLAG in Δ*mit1*-Mit1-FLAG cells and Prm1-HA in Δ*prm1*-Prm1-HA cells in glucose, glycerol, and methanol. Twenty micrograms of total protein was loaded into each *lane*, and the expression of β-actin was used as a positive control. Normalization of signal intensity to total protein loading is described under “Miscellaneous Methods.” *G*, a ChIP assay was performed with Δ*mit1*-Mit1-FLAG and Δ*prm1*-Prm1-HA cells grown in glucose, glycerol, and methanol media, respectively. Immunoprecipitation was conducted using anti-FLAG and anti-HA antibody, respectively. The data are expressed as binding (ChIP/input) for nine fragments of P*_AOX1_* relative to ChIP/input at the P*_ACT1_* region used as a reference. The *error bars* represent the standard deviation of three biological replicates, each with three technical replicates, assayed in duplicate. An independent-sample *t* test was used to determine statistical significance of the ChIP/input at P*_AOX1_* region groups relative to ChIP/input at P*_ACT1_* groups (ratios higher than 1 were considered) in corresponding samples. *, *p* < 0.05; **, *p* < 0.01. The nine fragments of P*_AOX1_* (−940 to −1 bp) are named A1–A9 in sequence with a 20-bp overlap between adjacent fragments and are depicted by a *solid* and an *empty rectangle* alternately under their names. The binding sites of Mxr1, Mit1, and Prm1 in *D* are also marked on the rectangles with the indicated colors.

To investigate the function of each Mit1 domain, we constructed strains expressing domain deletion mutants of Mit1. In addition, we constructed a strain in which Mit1 was replaced by Mpp1 (supplemental Table S1). All of these Mit1 or Mpp1 mutants were HA-tagged and were stably expressed in glycerol and methanol (Fig. 3*B*). The growth of and P*_AOX1_* activity in these strains were analyzed after exposure to methanol. As expected, complementation with Mpp1 restored the growth of and P*_AOX1_* activity in Δ*mit1* cells exposed to methanol (Fig. 3, *C–E*). This indicated that Mit1 and Mpp1 had similar functions following P*_AOX1_* activation, despite their low homology. Domain deletion analysis showed that ΔZF and ΔUR2 cells could not grow in the presence of methanol and that GFP expression in these cells decreased to ∼2 and 8%, respectively, compared with that in WT cells. These findings indicated that both ZF and UR2 are extremely important for Mit1 function. We have shown that the ZF domain of Mit1 is necessary for DNA binding (Fig. 4) and that the UR2 domain is necessary for its subcellular localization (Fig. 3*F*). ΔRR1, ΔUR1, and ΔUR4 cells showed growth defects in the presence of methanol, and P*_AOX1_* activity in these cells decreased to 88, 64, and 30%, respectively, compared with that in WT cells (Fig. 3, *C* and *E*). These results indicated that RR1, UR1, and UR4 also participated in P*_AOX1_* activation. ΔRR2 and ΔRR3 cells showed almost normal growth and P*_AOX1_* activity, indicating that RR2 and RR3 are not necessary for Mit1 function (Fig. 3, *C–E*). In contrast, ΔUR3 cells showed higher P*_AOX1_* activity but weaker growth than WT cells, suggesting that UR3 is not necessary for P*_AOX1_* activation but is necessary for the regulation of MUT pathway genes, which is important for growth in the presence of methanol (Fig. 3, *C–E*).

Interestingly, Δ*mit1*-Mpp1 cells showed remarkable Aox expression in the presence of glycerol (Fig. 3*D*), indicating that P*_AOX1_* overcame the glycerol-induced repression. GFP expression under the control of P*_AOX1_* in Δ*mit1*-Mpp1 cells exposed to glycerol was ∼4.8-fold and 5.8% of that in WT cells exposed to glycerol (basal expression) and methanol, respectively (Fig. 3*E*). These results indicated that Mit1 and Mpp1 developed different functions during evolution. In addition, ΔUR3 and ΔRR3 cells expressed Aox when exposed to glycerol (Fig. 3*D*). P*_AOX1_* activity in these cells was 3.6 and 2.4%, respectively, of that in WT cells exposed to methanol (Fig. 3*E*). This indicated that UR3 and RR3 may be involved in glycerol-induced repression of P*_AOX1_*. Because the three redundant regions of Mit1 were not present in *H. polymorpha* Mpp1, we constructed strains containing a double or triple deletion of RR1, RR2, and RR3 (*i.e.* ΔRR1ΔRR2, ΔRR1ΔRR3, ΔRR2ΔRR3, and ΔRR1ΔRR2ΔRR3 mutants). These cells showed only a slight growth defect in the presence of methanol (Fig. 3*C*). However, in the presence of glycerol, ΔRR1ΔRR3 cells showed strong Aox expression, whereas ΔRR2ΔRR3, ΔRR1ΔRR2ΔRR3, and ΔRR1ΔRR2 cells showed very weak or no Aox expression (Fig. 3*D*). The strength of P*_AOX1_* activity in these cells in the presence of glycerol was as follows: ΔRR1ΔRR3 (19%) > Δ*mit1*-Mpp1 (5.8%) > ΔUR3 (3.6%) > ΔRR3 (2.4%) > ΔRR1ΔRR2ΔRR3 (1.4%) (Fig. 3*D*). This result indicates that RR1, RR3, and UR3 were involved in the strict repression of P*_AOX1_* in the presence of glycerol. Therefore, we concluded that the structural dissimilarity between Mit1 and Mpp1 contributed to the differential repression of P*_AOX1_* and P*_MOX_* in the presence of glycerol.

##### Binding Sites of Mit1 on P_AOX1_ Are Different from Those of Mxr1 and Prm1

Because both Mit1 and Prm1 belong to the Zn(II)_2_Cys_6_ family, they may regulate P*_AOX1_* through direct binding. To verify this possibility, we performed EMSAs (*in vitro*) and ChIP assays (*in vivo*). In the EMSAs, peptides containing the ZF domains of Mit1 (aa 1–150) and Prm1 (aa 41–90) fused with a His_6_ tag were expressed in *E. coli* and purified by performing Ni^2+^-NTA affinity chromatography. The purified peptides were incubated with Cy5-labeled W1 (−940 to −704), W2 (−723 to −515), W3 (−534 to −367), and W4 (−386 to −162) fragments of P*_AOX1_*, with a 20-bp overlap between adjacent fragments. The results showed that the ZF domains of Mit1 and Prm1 could bind to P*_AOX1_ in vitro* (Fig. 4*A*). Interestingly, both Mit1 and Prm1 could bind to the W3 fragment of P*_AOX1_*. However, only Mit1 could bind to the W2 fragment and only Prm1 could bind to the W4 fragment of P*_AOX1_* (Fig. 4*A*). A DNase I footprinting assay was conducted using the P*_AOX1_* fragments shifted by Mit1 and Prm1 to determine the binding sites of Mit1 and Prm1 on these fragments. The results of this assay showed that Mit1 could bind to two regions on the W2 fragment, *i.e.* W2A (−717 to −696) and W2B (−688 to −673) and three regions on the W3 fragment, *i.e.* W3A (−520 to −503), W3B (−490 to −484), and W3C (−453 to −448) of P*_AOX1_* (Fig. 4*B*). Further, Prm1 could bind to W3D (−421 to −381) and W4A (−322 to −310) of P*_AOX1_* (Fig. 4*B*).

These results were verified by performing EMSAs. Results from the EMSAs showed that Mit1 could bind to W2 fragment lacking either W2A or W2B but could not bind to the W2 fragment lacking both W2A and W2B (Fig. 4*C*), indicating that Mit1 could bind to both W2A and W2B. Further, Mit1 could bind to W3 fragment lacking both W3B and W3C (Fig. 4*C*) but could not bind to the W3 fragment lacking W3A (Fig. 4*C*), indicating that Mit1 could positively bind to W3A. However, EMSAs did not show the binding of Mit1 to W3B and W3C, suggesting that it could weakly bind to these regions *in vitro*. Prm1 could not bind to W3 and W4 fragments lacking W3D and W4A, respectively, indicating that it could bind to regions W3D and W4A of P*_AOX1_* (Fig. 4*C*).

Because both Mit1 and Prm1 could bind to the W3 fragment, we determined whether they competed for binding sites on this fragment. To this end, we deleted W3D from the W3 fragment and added W3D to the 5′ terminus of the W4 fragment to produce the W3ΔD and W3D + W4 fragments, respectively. As expected, Mit1 could bind to the W3 fragment lacking W3D, but Prm1 could not (Fig. 4*C*). However, Mit1 could not bind to the W3D + W4 fragment (Fig. 4*C*). These results indicated that Mit1 and Prm1 bound to different sites on the W3 fragment. In addition, Mxr1 were proven to bind at six different sites of P*_AOX1_* containing core 5′-CYCC-3′ motif (20, 21) (Fig. 4*D*), and these sites were different from the binding sites of Mit1 and Prm1. Therefore, we concluded that Mit1, Prm1, and Mxr1 bound to different sites on P*_AOX1_* (Fig. 4*D*).

Next, we performed *in vivo* ChIP assay by using cells expressing Mit1-FLAG and Prm1-HA and exposed to glucose, glycerol, and methanol. Mit1-FLAG and Prm1-HA recovered the growth of Δ*mit1* and Δ*prm1* cells, respectively, in the presence of methanol (Fig. 4*E*), indicating that the fusion of Mit1 with FLAG and Prm1 with HA did not affect their function. Mit1-FLAG and Prm1-HA were detected in glucose-, glycerol-, and methanol-grown cells by Western blotting (Fig. 4*F*). P*_AOX1_* was divided into nine fragments (each about 120 bp) with a 20-bp overlap between adjacent fragments (Fig. 4*G*). Then nine pairs of primers corresponding to these fragments were designed to detect the immunoprecipitated DNA by quantitative PCR. Quantitative analysis of P*_AOX1_* enrichment showed that Mit1 and Prm1 preferably bound to the A3–A7 region of P*_AOX1_* (Fig. 4*G*). Moreover, Mit1 preferentially bound to A3 and A5, both of which contained the Mit1-binding sites concluded by EMSAs and DNase I footprinting assays (Fig. 4*G*). Prm1 preferentially bound to A5–A7, where Prm1-binding sites were distributed (Fig. 4*G*). In addition, the strengths of Mit1 and Prm1 binding to P*_AOX1_* were carbon-dependent. They became stronger gradually with the successive carbon source variation of glucose, glycerol, and methanol, whereas Prm1 showed no binding to P*_AOX1_* in glucose (Fig. 4*G*). Thus, Mit1 could bind to P*_AOX1_* in the presence of glucose, glycerol, and methanol. whereas Prm1 could bind to P*_AOX1_* in the presence of glycerol and methanol but not in the presence of glucose, and their binding sites were consistent with the results *in vitro*.

##### Mit1, Prm1, and Mxr1 Independently Activate P_AOX1_ in P. pastoris

**FIGURE 5.**
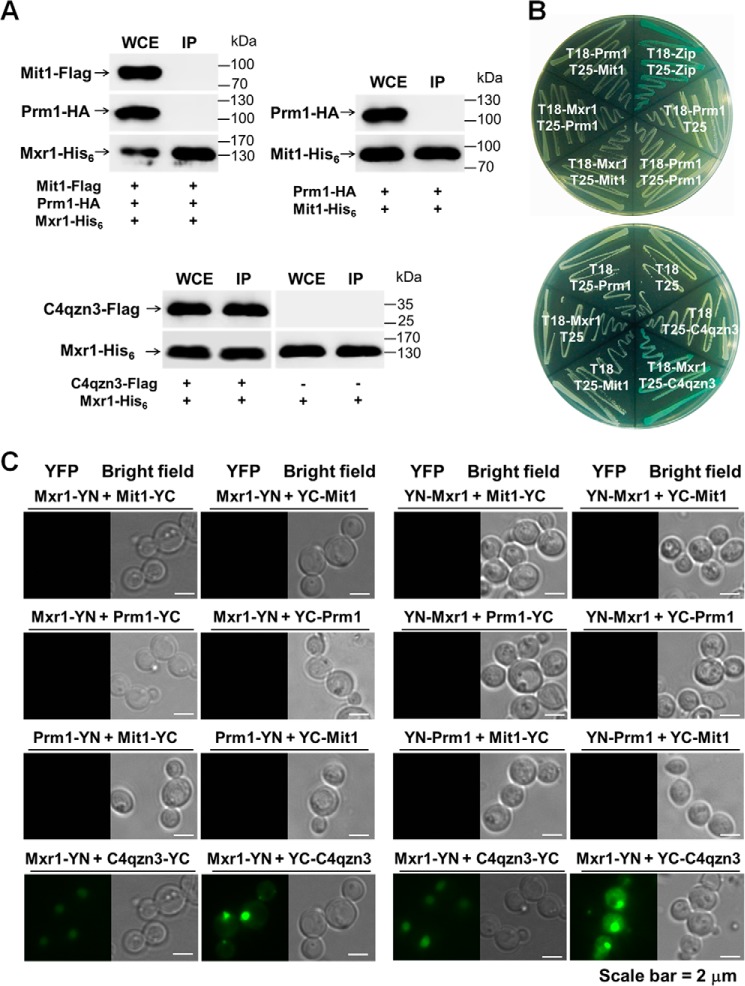
**Mit1, Prm1, and Mxr1 independently activate P*_AOX1_* in *P. pastoris*.** *A*, Ni^2+^-NTA pulldown assay of protein-protein interactions of Mit1, Prm1, and Mxr1. Mit1, Prm1, and Mxr1 fused with FLAG, HA, and His_6_ tags, respectively, were co-expressed in the WT strain. Cells were cultivated in methanol, and then whole cell proteins were extracted for the Ni^2+^-NTA pulldown assay. The His_6_-tagged proteins were pulled down by Ni^2+^-NTA beads. The pulled-down proteins were detected by Western blotting using antibodies of their specific tags. *WCE*, whole cell extract; *IP*, immunoprecipitated proteins. + and −, indicated fusion proteins were present or absent, respectively. The interaction between C4qzn3 and Mxr1 was used as a positive control (29). Three independent replicates were performed, and the pulled-down proteins were mixed together. Twenty micrograms of total protein was loaded into each whole cell extract lane. Twenty microliters of the same pulled-down protein samples was loaded into each immunoprecipitation lane in each experiment. Normalization of signal intensity to total protein loading is described under “Miscellaneous Methods.” *B*, B2H assay of protein-protein interactions of Mit1, Prm1, and Mxr1. Strains with proteins that could bind with each other showed up as a *blue colony* on LB plates supplemented with isopropyl 1-thio-β-d-galactopyranoside and β-galactosidase. T18 and T25 are two complementary fragments of adenylate cyclase (CyaA) from *Bordetella pertussis. Zip*, represents the leucine zipper motif of GCN4. Strains are indicated by their expressed T18 or T25 fusion proteins. A strain expressing T25-zip and T18-zip fusion proteins was used as a positive control. It showed up as a *blue colony* (Cya^+^ phenotype) as the dimerization of leucine zipper motifs appended to the T25 and T18 fragments. A strain expressing T18 and T25 was used as negative control. *C*, BiFC assay of protein-protein interactions of Mit1, Prm1, and Mxr1. Strains expressing protein partners fused with N-terminal aa 1–173 of YFP (*YN*) or C-terminal aa 155–239 of YFP (*YC*) are indicated on each *panel*. The protein-protein interactions of Mit1, Prm1, and Mxr1 were tested in methanol, and the interactions of Mxr1 and C4qzn3 were tested in glucose (*bottom left*) and ethanol (*bottom right*). Positive interactions resulted in fluorescence.

Mit1, Prm1, and Mxr1 are necessary for the activation of P*_AOX1_*. Deletion of either of these transcription factors dramatically decreases P*_AOX1_* activity to <10% of that in WT cells and inhibits the growth of cells in the presence of methanol. Therefore, we examined whether Mit1, Prm1, and Mxr1 function by forming heterodimers. To this end, we performed a Ni^2+^-NTA pulldown assay. We constructed a WT-MPX strain coexpressing Mit1-FLAG, Prm1-HA, and Mxr1-His_6_ and a WT-PM strain coexpressing Prm1-HA and Mit1-His_6_. Interaction between Mxr1 and C4qzn3 (14-3-3 protein, also called Bmh1) was determined as a positive control (29). These strains were exposed to methanol, and their whole cell lysates were extracted. His_6_-tagged proteins were pulled down by using Ni^2+^-NTA beads. The proteins obtained were analyzed by performing Western blotting with antibodies against the specific tags. Results of the Western blotting showed no interactions among the examined transcription factors (Fig. 5*A*). These results were confirmed by performing a B2H assay and a BiFC assay (Fig. 5, *B* and *C*). Thus, our results indicated that Mit1, Prm1, and Mxr1 activated P*_AOX1_* independently and not by forming heterodimers.

##### Mit1 Is Inductively Expressed whereas Prm1 Is Constitutively Expressed in the Presence of Methanol, and Their Functions Are Not Related to Their Localization

**FIGURE 6.**
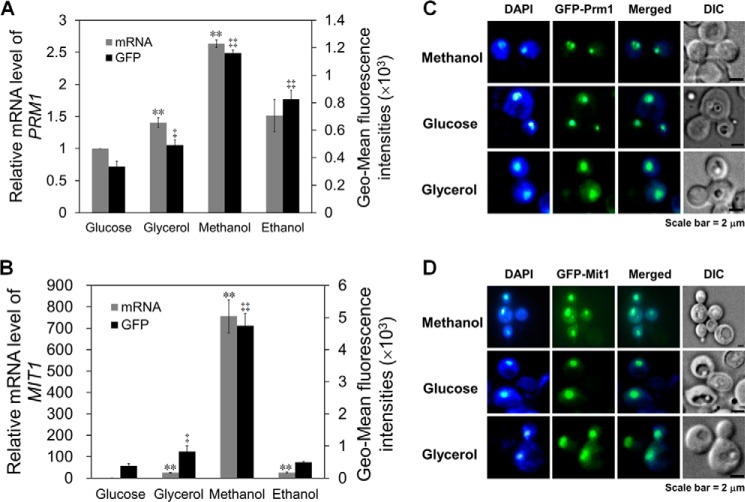
**Regulatory profiles and localizations of Mit1 and Prm1 in different carbon sources.** *A*, transcription level of *PRM1* and expression of GFP driven by P*_PRM1_* in WT cells cultured in glucose, glycerol, methanol, and ethanol. *B*, transcription level of *MIT1* and expression of GFP driven by P*_MIT1_* in the WT cells cultured in glucose, glycerol, methanol, and ethanol. For *A* and *B*, the mRNA levels were normalized to the levels of the mRNA of housekeeping gene *ACT1* in each sample. The relative expression level indicated on the *y* axis (2^−ΔΔCT^) for each gene at different carbon sources was normalized for its expression in glucose-grown cells. The *error bars* represent the standard deviation of three biological replicates, each with three technical replicates, assayed in duplicate. An independent-sample *t* test was used to determine the statistical significance of the glycerol, methanol, and ethanol groups relative to glucose groups in the corresponding mRNA or GFP assay. mRNA: **, *p* < 0.01. GFP: ‡, *p* < 0.05; ‡‡, *p* < 0.01. *C* and *D*, localization of GFP-Prm1 (*C*) and GFP-Mit1 (*D*) in glucose, glycerol, and methanol. DAPI was used to stain the cell nucleus. *DIC*, differential interference contrast.

To investigate the regulatory profiles of *MIT1* and *PRM1*, their expression levels were measured by assessing their transcription and expression of GFP under the control of their specific promoters in the presence of glucose, glycerol, methanol, and ethanol. Minimal changes (<2.6-fold) were observed in the mRNA level of *PRM1* in cells exposed to glucose, glycerol, methanol, and ethanol (Fig. 6*A*). However, the mRNA level of *MIT1* increased dramatically (up to 750-fold) in cells exposed to methanol (Fig. 6*B*). Expression levels of GFP under the control of P*_MIT1_* and P*_PRM1_* were similar to those of *MIT1* and *PRM1*. Therefore, we concluded that Mit1 was inductively expressed in the presence of methanol, whereas Prm1 was almost constitutively expressed in the presence of glucose, glycerol, methanol, and ethanol.

Mxr1 is localized to the cytoplasm in cells exposed to glucose and is translocated to the nucleus in cells exposed to glycerol, methanol, or oleate (16). To determine whether the regulation of Mit1 followed a similar pattern, its localization was investigated using Prm1 as a control. WT cells were transfected with vectors expressing GFP-Mit1 or GFP-Prm1 under the control of P*_GAP_*. The results showed that both GFP-Mit1 and GFP-Prm1 were localized to the nucleus in the presence of glucose, glycerol, and methanol (Fig. 6, *C* and *D*), indicating that their function was independent of their subcellular localization.

##### Methanol Induction Signal Transducing to Mit1 is Mediated by Prm1

**FIGURE 7.**
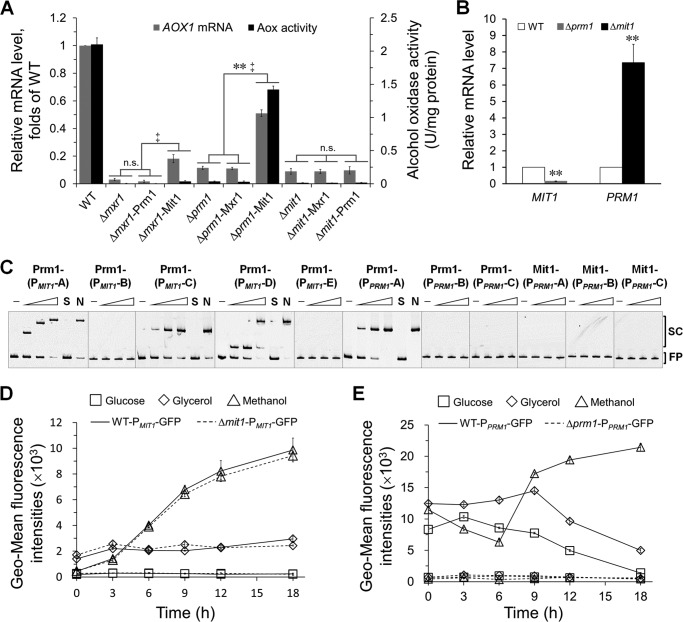
**Derepression/induction of *AOX1* promoter by Mit1, Prm1, and Mxr1.** *A*, relative mRNA levels of *AOX1* and enzyme activity of Aox in mutant strains. The mRNA levels were normalized to that of the housekeeping gene *ACT1* in each sample. The relative expression level indicated on the *y* axis (2^−CT^) for each gene was normalized to that in the WT strain grown in methanol. The enzyme activity of Aox was assayed as described previously (37, 38). A unit of Aox represents 1 μmol of product min^−1^ mg^−1^ of protein at 30 °C. The *error bars* represent the standard deviation of three biological replicates, each with three technical replicates, assayed in duplicate. An independent-sample *t* test was used to determine the statistical significance of different groups as indicated. *AOX1* mRNA: **, *p* < 0.01. Aox activity: ‡, *p* < 0.05; *n.s*., no significance. *B*, detection of mRNA levels of *PRM1* in the Δ*mit1* mutant and *MIT1* in the Δ*prm1* mutant in methanol. Other *labeling* is the same as in *A*. An independent-sample *t* test was used to determine the statistical significance of mutant groups relative to WT groups for corresponding gene. **, *p* < 0.01. *C*, EMSAs of *P. pastoris* Prm1 or Mit1 with fragments of the *MIT1* promoter (P*_MIT1_*) and the *PRM1* promoter (P*_PRM1_*). EMSAs were performed by incubating Cy5-labeled P*_MIT1_*-A (−1,000 to −701 bp of *MIT1*), P*_MIT1_*-B (−800 to −601 bp of *MIT1*), P*_MIT1_*-C (−700 to −351 bp of *MIT1*), P*_MIT1_*-D (−450 to −251 bp of *MIT1*), P*_MIT1_*-E (−350 to −1 bp of *MIT1*), P*_PRM1_*-A (−699 to −540 bp of *PRM1*), P*_PRM1_*-B (−562 to −314 bp of *PRM1*), and P*_PRM1_*-C (−338 to −112 bp of *PRM1*) with the zinc finger domains of *P. pastoris* Prm1 (aa 41–90) or Mit1 (aa 1–150) expressed by *E. coli*. The experimental procedure and other labeling were the same as described in the legend for Fig. 4*A. D* and *E*, detection of self-regulation of Mit1 (*D*) and Prm1 (*E*). GFP expressed by P*_MIT1_* and P*_PRM1_* was used to quantify the self-regulation of Mit1 and Prm1 in the strains of WT-P*_MIT1_*-GFP, Δ*mit1*-P*_MIT1_*-GFP, WT-P*_PRM1_*-GFP, and Δ*prm1*-P*_PRM1_*-GFP. GFP expression was analyzed as described in the legend for Fig. 2.

The above results and the results of previous studies (16, 18, 19) indicated that Mit1, Prm1, and Mxr1 are extremely important for activating P*_AOX1_*. This suggested that the signal for methanol induction may be transmitted through a cascade mechanism involving these regulators. Deletion of either of these factors may disrupt this signal transduction cascade, and overexpression of downstream regulators may restore the transmission of the methanol signal to P*_AOX1_*. Thus, a series of strains with one regulator deletion and another overexpression was constructed. Western blotting and quantitative PCR confirmed the overexpression of these factors in these strains (data not shown). Mxr1 (or its homolog, Trm2) involved in the utilization of various carbons and was necessary for the derepression of P*_AOX1_* (13, 16, 29, 30). In the absence of *MXR1*, overexpression of Prm1 or Mit1 did not significantly rescue P*_AOX1_* activity, despite a small increase in *AOX1* mRNA expression and Aox activity in Δ*mxr1*-Mit1 cells (Fig. 7*A*). This suggested that Mxr1-dependent derepression occurred before methanol-specific activation of P*_AOX1_*. In Δ*prm1*-Mit1 cells (*PRM1* deletion mutants overexpressing Mit1), the mRNA level of *MIT1* was 20% of that in WT cells (data not shown), whereas the mRNA level of *AOX1* and activity of Aox were 51 and 68%, respectively, of those in WT cells (Fig. 7*A*). These results indicated that Mit1 acted downstream of Prm1 to activate P*_AOX1_*. Next, we analyzed the transcription of *PRM1* in Δ*mit1* cells and *MIT1* in Δ*prm1* cells and compared it with the transcription of these genes in WT cells exposed to methanol. The transcription level of *MIT1* in Δ*prm1* cells was ∼0.15-fold of that in WT cells, whereas that of *PRM1* in Δ*mit1* cells was ∼7-fold of that in WT cells (Fig. 7*B*). This indicated that Prm1 induced *MIT1* expression and Mit1 could repress *PRM1* expression. The results of EMSAs showed that Prm1 regulated P*_MIT1_* by directly binding its P*_MIT1_*-A (−1000 to −701 bp), P*_MIT1_*-C (−700 to −351 bp), and P*_MIT1_*-D (−450 to −251 bp) regions, but Mit1 did not bind to P*_PRM1_* (Fig. 7*C*).

Some genes are regulated by the proteins that they encode, *e.g.* Trm1 binds to its native promoter in *C. boidinii* exposed to glucose and methanol (17). Mit1 and Prm1 may also regulate their native promoters. Therefore, we quantified the activities of P*_MIT1_* in Δ*mit1* cells and P*_PRM1_* in Δ*prm1* cells using GFP as a reporter. GFP expression driven by P*_PRM1_* decreased in Δ*prm1* cells, whereas GFP expression driven by P*_MIT1_* remained unchanged in Δ*mit1* cells exposed to glucose, glycerol, or methanol compared with that in WT cells (Fig. 7, *D* and *E*). The EMSA results confirmed that Prm1 could bind to its native promoter at region P*_PRM1_*-A (−699 to −540 bp of *PRM1*) (Fig. 7*C*). These results proved that Prm1 regulates its native promoter by direct binding.

**FIGURE 8.**
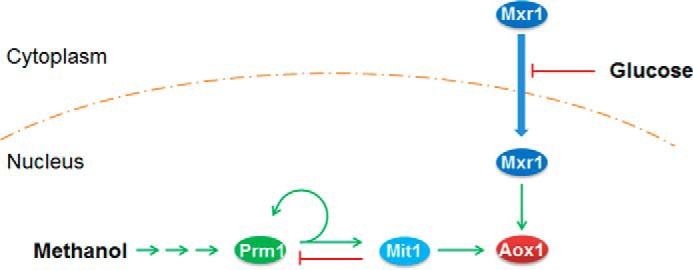
**Regulatory model of P*_AOX1_* activation by Mxr1, Prm1, and Mit1.** When glucose existed, P*_AOX1_* was repressed because Mxr1 located to cytoplasm and failed to show the derepression function. When methanol was used as the sole carbon source, Mxr1 was transported to the nucleus and relieved the repression of P*_AOX1_*. The methanol induction signal transmitted from Prm1 to Mit1 and then large amounts of Mit1 induced the strong expression of P*_AOX1_*. During this progression, Prm1 induced the expression of itself and Mit1, and Mit1 repressed the expression of Prm1.

Therefore, we proposed a regulatory model of P*_AOX1_* activation by methanol (Fig. 8). In cells exposed to glucose, Mxr1 was localized to the cytoplasm, which resulted in the strong repression of P*_AOX1_*. However, in cells exposed to methanol as the sole carbon source, Mxr1 was translocated to the nucleus, which resulted in the derepression of P*_AOX1_*. Thus, methanol induction signal could transmit to Prm1, which induced its self-expression and also expression of Mit1. Activated Mit1 acted downstream of Prm1 and strongly induced P*_AOX1_*. As a part of the feedback loop, Mit1 inhibited the expression of Prm1 to prevent its own accumulation.

## Discussion

In this study, we determined the function of transcription activator Mit1 and elucidated its relationship with Mxr1 and Prm1 in *P. pastoris*. Similar to Mpp1 (a homolog of Mit1 in *H. polymorpha*), Mit1 belongs to the Zn(II)_2_Cys_6_ family and plays a critical role in methanol metabolism (15). Exposure of *H. polymorpha* Δ*mpp1* cells to methanol strongly decreased the expression of various peroxin-encoding genes (*PEX3*, *PEX5*, and *PEX10*) involved in peroxisome formation (15). However, this was not observed in *P. pastoris* Δ*mit1* cells. Mpp1 was found to be involved in peroxisome proliferation, but Mit1 was not, indicating that these two proteins regulate peroxisome proliferation differently despite being homologs. Moreover, complementation of *P. pastoris* Δ*mit1* cells with *H. polymorpha* Mpp1 relieved the repression of P*_AOX1_* in the presence of glycerol. Structural analysis indicated that many regions of Mit1 showed low homology to those of Mpp1, with three redundant regions being absent in Mpp1. Domain deletion analysis indicated that the RR1, RR2, RR3, and UR3 domains participated in glycerol-induced repression of P*_AOX1_*. The function of the RR3 domain may be complemented by the RR1 domain but is weakened by the RR2 domain, because the derepression level in ΔRR3 cells was weaker than that in ΔRR1ΔRR3 cells but was stronger than that in ΔRR2ΔRR3 cells. Moreover, the dissimilarity in UR3 or other domains may contribute to the glycerol-induced repression of P*_AOX1_* because the derepression level in Δ*mit1*-Mpp1 cells was stronger than that in ΔRR1ΔRR2ΔRR3 cells. This is the first study to verify the transcription factors involved in regulating species-specific derepression of P*_AOX1_* in the presence of glycerol.

Xuan *et al.* (23) showed that two regions of P*_AOX1_* (region D, −638 to −510 bp, and region E, −552 to −442 bp) were extremely important for its activity. Deletion of these regions decreased P*_AOX1_* activity to 16 and 14%, respectively, compared with that of the WT promoter. We observed that region D included two Mxr1-binding sites and a part of Mit1-binding site W3A, whereas region E included W3A, indicating that the binding of Mit1 to W3A was extremely important for activating P*_AOX1_*. Deletion of regions G (−441 to −361 bp) and H (−391 to −311 bp), which contain Prm1-binding sites, maintained the P*_AOX1_* activity at 58 and 55%, respectively (23). Impaired P*_AOX1_* activity in Δ*prm1* cells was rescued by restoring Mit1 expression (Fig. 7*A*). These results indicated that Mit1 is more important for P*_AOX1_* activation than Prm1.

An *in vivo* assay showed that the binding of Mit1 and Prm1 to P*_AOX1_* was carbon-dependent, and the binding strengths were weak (or none) in glucose, mild in glycerol, but strong in methanol. Binding of Prm1 to P*_AOX1_* might be repressed by glucose-related repressors or post-transcriptional modification of Prm1 by kinases or phosphatases. In contrast, Sahu *et al.* (19) found that 220 N-terminal amino acids of Prm1, including the ZF domain, did not bind to P*_AOX1_ in vitro*. This may be because the Prm1 ZF domain-containing peptide used in the present study was only 50 aa long, which is much shorter than the 220-aa-long peptide used by Sahu *et al.* (19). The large peptide of Prm1 may contain a complex protein structure that interferes with its binding to P*_AOX1_*. In *H. polymorpha*, Mpp1 activates P*_MOX_*; however, it is unclear whether Mpp1 binds to P*_MOX_* (15). In *C. boidinii*, Trm1 binds to P*_AOD1_* in the presence of methanol but not in the presence of glucose; however, it is unclear whether this binding occurs in the presence of glycerol (17). Here, we determined the specific binding sites of Mit1 and Prm1 in P*_AOX1_*, which differed from Mxr1-binding sites containing the core 5′-CYCC-3′ motif (21, 30). Our results may be useful in performing relative studies on these methylotrophic yeasts.

Nonfermentative metabolism in *Saccharomyces cerevisiae* is generally regulated by complexes containing different transcription factors (31). An Ni^2+^-NTA pulldown assay, a B2H assay, and a BiFC assay showed no interaction among Mit1, Prm1, and Mxr1 (Fig. 5). However, these three transcription factors were essential for regulating P*_AOX1_* activation. In addition, a yeast two-hybrid assay was also performed. Unfortunately, when Mit1, Prm1, or Mxr1 was fused with the GAL4-binding domain, the reporter genes were activated, indicating that Mit1, Prm1, or Mxr1 itself was enough to active the promoter without the GAL4-activating domain (data not shown). Therefore, we suggest that the three factors bound to P*_AOX1_* independently and might synergistically recruit regulatory machinery.

Deletion of Mit1, Prm1, or Mxr1 inhibited the transmission of the methanol induction signal to P*_AOX1_*. We observed that the methanol induction signal was transmitted among these regulators through a cascade. Mxr1 is localized to the cytoplasm of cells exposed to glucose but is translocated to the nucleus of cells exposed to glycerol, ethanol, and methanol (16). This indicates that the glucose-induced repression of P*_AOX1_* might be regulated by subcellular localization variation of Mxr1. Other studies have also shown that Mxr1 or its homolog, Trm2, derepresses methanol-inducible promoters (13, 16, 29). In fact, there is no explicit boundary between derepression and induction, and Mxr1 may be involved in both of these processes. Our results indicated that Mxr1 is mainly involved and is essential for the derepression of P*_AOX1_*. This function of Mxr1 was different from that of Mit1 and Prm1, which mainly responded to methanol induction (Fig. 7*A*). Deletion of *MXR1* blocked the methanol induction signal because of the failure of derepression. Accordingly, overexpression of Mit1 or Prm1 could not improve Aox expression (Fig. 7*A*). During methanol induction, the methanol signal was transmitted to P*_MIT1_*, resulting in dramatic Mit1 expression (Fig. 6*B*). Further studies indicated that Prm1 transmitted the methanol induction signal to P*_MIT1_*. Because cells exposed to glucose, glycerol, and methanol showed sustained Prm1 expression (Fig. 6*A*), it is possible that Prm1 transmitted the methanol induction signal by altering its structure rather than by altering the dose of the signal.

Regulation of *P. pastoris* P*_AOX1_* by Mxr1, Prm1, and Mit1 is similar to the induction of oleate response genes meditated by Adr1, Oaf1, and Pip2 (32–34). The regulators of these two pathways have many common features. Adr1 and Mxr1 are homologs. Prm1 and Oaf1 are constitutively expressed, whereas Mit1 and Pip2 are inductively expressed by methanol and oleate, respectively. Moreover, *AOX1* and oleate-response genes are repressed by glucose and are induced by methanol and oleate, respectively. In low glucose condition, Adr1 and Mxr1 derepress oleate-response genes and genes involved in the MUT pathway, respectively (13, 29, 32). In the presence of oleate or methanol, Oaf1 and Prm1 bind to P*_PIP2_* and P*_MIT1_*, respectively, leading to their activation. Finally, Pip2, Oaf1, and Adr1 bind to the promoters of oleate-response genes, and Mit1, Prm1, and Mxr1 bind to P*_AOX1_* (35). These factors then recruit transcriptional machinery to initiate gene expression. However, Oaf1 and Pip2 form heterodimers (32), whereas Prm1 and Mit1 do not form heterodimers, as observed in the present study. Further, oleate activated Oaf1 by binding directly to it (36). However, it is unclear how methanol activates Prm1.

In conclusion, we have identified Mit1, a critical transcription factor of P*_AOX1_*, in which the structure and function differ from those of its heterologous homolog, Mpp1. Furthermore, we determined that the methanol induction signal was transduced through a cascade in which Mit1 functions downstream and tightly regulates P*_AOX1_*. These results may be useful in elucidating the mechanisms that underlie the response of methylotrophic yeasts to methanol, in rewiring the methanol induction circuit for improving P*_AOX1_* activity, and in developing a novel methanol-free *Pichia* expression system for application in the pharmaceutical and food industries.

## Author Contributions

M. C. and X. Z. conceived and coordinated the study. All the experiments in this paper were designed, performed, and analyzed by X. W., Q. W., and M. C.. X. W. conducted most of the experiments. Q. W. conducted the EMSAs and DNase I footprinting assays. P. B., J. W., L. S., and W. S. contributed to the discussion and preparation of some experiments. X. W. wrote the paper with revisions made by M. C. M. Z. and Y. Z. participated in making the revisions. All authors reviewed the results and approved the final version of the manuscript.

## Supplementary Material

Supplemental Data

## Footnotes

2 The abbreviations used are:

MUT: methanol utilization
aa: amino acid
Aox: alcohol oxidase
*AOX1*TT: *AOX1* terminator
FAM: 6-carboxyfluorescein
BiFC: bimolecular fluorescence complementation
B2H: bacterial two-hybrid
ZF: zinc finger
BFP: blue fluorescent protein
NTA: nitrilotriacetic acid
RR: redundant region
UR: undefined region.
