# Supplementary material for: Mit1 Transcription Factor Mediates Methanol Signaling and Regulates the Alcohol Oxidase 1 (AOX1) Promoter in Pichia pastoris
Source: J Biol Chem. 2016 Jan 31;291(12):6245–61. doi: 10.1074/jbc.M115.692053 (PMC4813576; doi:10.1074/jbc.M115.692053)
Supplement: Supplemental Data [file supp_291_12_6245__index.html]

Mit1 Transcription Factor Mediates Methanol Signaling and Regulates the Alcohol Oxidase 1 (AOX1) Promoter in Pichia pastoris — Mit1 Acts Downstream and Regulates AOX1 Promoter — Supplemental Data 

# Mit1 Transcription Factor Mediates Methanol Signaling and Regulates the Alcohol Oxidase 1 (*AOX1*) Promoter in *Pichia pastoris*

## Supplemental Data

**Files in this Data Supplement:**

- Supplemental Information
