# Supplementary material for: Mit1 Transcription Factor Mediates Methanol Signaling and Regulates the Alcohol Oxidase 1 (AOX1) Promoter in Pichia pastoris
Source: J Biol Chem. 2016 Jan 31;291(12):6245–61. doi: 10.1074/jbc.M115.692053 (PMC4813576; doi:10.1074/jbc.M115.692053)
Supplement: Supplemental Data [file supp_M115.692053_jbc.M115.692053-1.docx]

**Supplemental Information**

Mit1 Transcription Factor Mediates Methanol Signaling and Regulates Alcohol Oxidase 1 (*AOX1*) Promoter in *Pichia pastoris**

**Xiaolong Wang^1^, Qi Wang^1^, Jinjia Wang^1^, Peng Bai^1^, Lei Shi^1^, Wei Shen^1^, Mian Zhou^1^, Xiangshan Zhou^1^, Yuanxing Zhang^1,2^ and Menghao Cai^1^**

^1^ State Key Laboratory of Bioreactor Engineering, East China University of Science and Technology, Shanghai 200237, China

^2^ Shanghai Collaborative Innovation Center for Biomanufacturing (SCICB), Shanghai 200237, China

*Running title: *Mit1 acts downstream and regulates AOX1 promoter*

To whom correspondence should be addressed: Menghao Cai, State Key Laboratory of Bioreactor Engineering, East China University of Science and Technology, 130 Meilong Road, Shanghai 200237, China, Tel.: (86) 21-64253065; Fax: (86) 21-64253025; E-mail: cmh022199@ecust.edu.cn.

**Table S1** Strains used in this study.

| Strain | Genotype | Source |
| --- | --- | --- |
| ***E. coli*** |  |  |
| Top 10 | F^-^[*lacI*^q^ Tn*10*(Tet^r^)] *mcrA* Φ80*lacZ* ΔM15 Δ*lac X74* *deoR* *recA1* | Invitrogen |
| BL21 (DE3) | F^-^ ompT hsdS(r_B_^-^m_B_^-^) gal dcm(DE3) | Invitrogen |
| *BTH101* | F^−^ cya-99 araD139 galE15 galK16 rpsL1 (Strr) hsdR2 mcrA1 mcrB1 | Euromedex |
| T18/T25 | *BTH101* harboring pUT18C and pKT25 | This study |
| T18-zip/T25-zip | *BTH101* harboring pUT18C-zip and pKT25-zip | This study |
| T18-Prm1/T25 | *BTH101* harboring pUT18C-Prm1 and pKT25 | This study |
| T18-Mxr1/T25 | *BTH101* harboring pUT18C-Mxr1 and pKT25 | This study |
| T18/T25-Mit1 | *BTH101* harboring pUT18C and pKT25-Mit1 | This study |
| T18/T25-Prm1 | *BTH101* harboring pUT18C and pKT25-Prm1 | This study |
| T18/T25-Mxr1 | *BTH101* harboring pUT18C and pKT25-Mxr1 | This study |
| T18/T25-C4qzn3 | *BTH101* harboring pUT18C and pKT25-C4qzn3 | This study |
| T18-Prm1/T25-Mit1 | *BTH101* harboring pUT18C-Prm1 and pKT25-Mit1 | This study |
| T18-Prm1/T25-Prm1 | *BTH101* harboring pUT18C-Prm1 and pKT25-Prm1 | This study |
| T18-Prm1/T25-Mxr1 | *BTH101* harboring pUT18C-Prm1 and pKT25-Mxr1 | This study |
| T18-Mxr1/T25-Mit1 | *BTH101* harboring pUT18C-Mxr1 and pKT25-Mit1 | This study |
| T18-Mxr1/T25-Prm1 | *BTH101* harboring pUT18C-Mxr1 and pKT25-Prm1 | This study |
| T18-Mxr1/T25-C4qzn3 | *BTH101* harboring pUT18C-Mxr1 and pKT25-C4qzn3 | This study |
| ***H. polymorpha*** |  |  |
| CBS4732 | Wild type | CGMCC |
| ***P. pastoris*** |  |  |
| GS115 | *his4* | Invitrogen |
| Δ*mit1* | GS115 *mit1*Δ::*hph his4* | This study |
| Δ*prm1* | GS115 *prm1*Δ::*Sh ble his4* | This study |
| Δ*mxr1* | GS115 *mxr1*Δ::*Sh ble his4* | This study |
| Δ*mit1*-Mit1 | Δ*mit1* P*_MIT1_*::pGMM1 (P*_MIT1_*-Mit1 *Sh ble*) | This study |
| Δ*prm1*-Prm1 | Δ*prm1* P*_PRM1_*::pAPP1 (P*_PRM1_*-Prm1 *hph*) | This study |
| Δ*mxr1*-Mxr1 | Δ*mxr1* *his4*::pPXX1 (P*_MXR1_*-Mxr1 *HIS4, KAN*) | This study |
| WT-GMit1 | GS115 P*_GAP_*::pGGLMit1 (P*_GAP_*-GFP–Mit1 *Sh ble*) | This study |
| WT-GPrm1 | GS115 P*_GAP_*::pGGLPrm1 (P*_GAP_*-GFP–Prm1 *Sh ble*) | This study |
| WTA | GS115 *his4*::pP-GFP (P*_AOX1_*-GFP *HIS4, KAN*) | This study |
| Δ*mit1*A | Δ*mit1* *his4*::pP-GFP (P*_AOX1_*-GFP *HIS4, KAN*) | This study |
| Δ*prm1*A | Δ*prm1* *his4*::pP-GFP (P*_AOX1_*-GFP *HIS4, KAN*) | This study |
| Δ*mxr1*A | Δ*mxr1* *his4*::pP-GFP (P*_AOX1_*-GFP *HIS4, KAN*) | This study |
| WTG | GS115 *his4*::pPGG (P*_GAP_*-GFP *HIS4, KAN*) | This study |
| Δ*mit1*G | Δ*mit1* *his4*::pPGG (P*_GAP_*-GFP *HIS4, KAN*) | This study |
| Δ*prm1*G | Δ*prm1* *his4*::pPGG (P*_GAP_*-GFP *HIS4, KAN*) | This study |
| WT-P*_MIT1_*-GFP | GS115 P*_MIT1_*::pGMG (P*_MIT1_*-GFP *Sh ble*) | This study |
| WT-P*_PRM1_*-GFP | GS115 P*_PRM1_*::pAPG (P*_PRM1_*-GFP *hph*) | This study |
| Δ*mit1*-P*_MIT1_*-GFP | Δ*mit1* P*_MIT1_*::pGMG (P*_MIT1_*-GFP *Sh ble*) | This study |
| Δ*prm1*-P*_PRM1_*-GFP | Δ*prm1* P*_PRM1_*::pAPG (P*_PRM1_*-GFP *hph*) | This study |
| WT-BFP–SKL | GS115 *his4*::pPWBS (P*_GCW14_*-BFP–SKL *HIS4, KAN*) | This study |
| Δ*mit1*-BFP–SKL | Δ*mit1* *his4*::pPWBS (P*_GCW14_*-BFP–SKL *HIS4, KAN*) | This study |
| Δ*prm1*-BFP–SKL | Δ*prm1* *his4*::pPWBS (P*_GCW14_*-BFP–SKL *HIS4, KAN*) | This study |
| Δ*mxr1*-BFP–SKL | Δ*mxr1* *his4*::pPWBS (P*_GCW14_*-BFP–SKL *HIS4, KAN*) | This study |
| Δ*mit1*-Mpp1 | Δ*mit1* P*_MIT1_*::pGM-Mpp1 (P*_MIT1_*-Mpp1–Flag *Sh ble*) | This study |
| Δ*mit1*A-Mpp1 | Δ*mit1*A P*_MIT1_*::pGM-Mpp1 (P*_MIT1_*-Mpp1–Flag *Sh ble*) | This study |
| ΔZF | Δ*mit1* *his4*::pP-GFP (P*_AOX1_*-GFP *HIS4*) P*_MIT1_*::pGMMΔZF (P*_MIT1_*-Mit1ΔZF–Flag *Sh ble*) | This study |
| ΔRR1 | Δ*mit1* *his4*::pP-GFP (P*_AOX1_*-GFP *HIS4*) P*_MIT1_*::pGMMΔRR1 (P*_MIT1_*-*MIT1*ΔRR1–Flag *Sh ble*) | This study |
| ΔUR1 | Δ*mit1* *his4*::pP-GFP (P*_AOX1_*-GFP *HIS4*) P*_MIT1_*::pGMMΔUR1 (P*_MIT1_*-Mit1ΔUR1–Flag *Sh ble*) | This study |
| ΔRR2 | Δ*mit1* *his4*::pP-GFP (P*_AOX1_*-GFP *HIS4*) P*_MIT1_*::pGMMΔRR2 (P*_MIT1_*-Mit1ΔRR2–Flag *Sh ble*) | This study |
| ΔUR2 | Δ*mit1* *his4*::pP-GFP (P*_AOX1_*-GFP *HIS4*) P*_MIT1_*::pGMMΔUR2 (P*_MIT1_*-Mit1ΔUR2–Flag *Sh ble*) | This study |
| ΔUR3 | Δ*mit1* *his4*::pP-GFP (P*_AOX1_*-GFP *HIS4*) P*_MIT1_*::pGMMΔUR3 (P*_MIT1_*-Mit1ΔUR3–Flag *Sh ble*) | This study |
| ΔRR3 | Δ*mit1* *his4*::pP-GFP (P*_AOX1_*-GFP *HIS4*) P*_MIT1_*::pGMMΔRR3 (P*_MIT1_*-Mit1ΔRR3–Flag *Sh ble*) | This study |
| ΔUR4 | Δ*mit1* *his4*::pP-GFP (P*_AOX1_*-GFP *HIS4*) P*_MIT1_*::pGMMΔUR4 (P*_MIT1_*-Mit1ΔUR4–Flag *Sh ble*) | This study |
| ΔRR1ΔRR2 | Δ*mit1* *his4*::pP-GFP (P*_AOX1_*-GFP *HIS4*) P*_MIT1_*::pGMMΔRR1ΔRR2 (P*_MIT1_*-Mit1ΔRR1ΔRR2–Flag *Sh ble*) | This study |
| ΔRR1ΔRR3 | Δ*mit1* *his4*::pP-GFP (P*_AOX1_*-GFP *HIS4*) P*_MIT1_*::pGMMΔRR1ΔRR3 (P*_MIT1_*-Mit1ΔRR1ΔRR3–Flag *Sh ble*) | This study |
| ΔRR2ΔRR3 | Δ*mit1* *his4*::pP-GFP (P*_AOX1_*-GFP *HIS4*) P*_MIT1_*::pGMMΔRR2ΔRR3 (P*_MIT1_*-Mit1ΔRR2ΔRR3–Flag *Sh ble*) | This study |
| ΔRR1ΔRR2ΔRR3 | Δ*mit1* *his4*::pP-GFP (P*_AOX1_*-GFP *HIS4*) P*_MIT1_*::pGMMΔRR1ΔRR2ΔRR3 (P*_MIT1_*-Mit1ΔRR1ΔRR2ΔRR3  –Flag *Sh ble*) | This study |
| Δ*mit1*-Mxr1 | Δ*mit1* P*_GAP_*::pP6GX1 (P*_GAP_*-Mxr1–HA *Blasticidin*) | This study |
| Δ*prm1*-Mxr1 | Δ*prm1* P*_GAP_*::pP6GX1 (P*_GAP_*-Mxr1–HA *Blasticidin*) | This study |
| Δ*mit1*-Prm1 | Δ*mit1* P*_GAP_*::pPGP1 (P*_GAP_*-Prm1–HA *HIS4, KAN*) | This study |
| Δ*mxr1*-Prm1 | Δ*mxr1* P*_GAP_*::pPGP1 (P*_GAP_*-Prm1–HA *HIS4, KAN*) | This study |
| Δ*prm1*-Mit1 | Δ*prm1* P*_GAP_*::pAGM1 (P*_GAP_*-Mit1–HA *hph*) | This study |
| Δ*mxr1*-Mit1 | Δ*mxr1* P*_GAP_*::pAGM1 (P*_GAP_*-Mit1–HA *hph*) | This study |
| Δ*mit1*-Mit1–Flag | Δ*mit1* P*_MIT1_*:pGMM1Flag (P*_MIT1_*-Mit1–Flag *Sh ble*) | This study |
| Δ*prm1*-Prm1–HA | Δ*prm1* P*_PRM1_*::pPPP1HA (P*_PRM1_*-Prm1–HA *HIS4, KAN*) | This study |
| WT-MPX | GS115 P*_GAP_*::pGGM1Flag (P*_GAP_*-Mit1–Flag *Sh ble*) P*_PRM1_*::pPPP1HA (P*_PRM1_*-Prm1–HA *HIS4, KAN*) P*_MXR1_*::pAXX1His_6_ (P*_MXR1_*-Mxr1–His_6_ *hph*) | This study |
| WT-XB | GS115 P*_MXR1_*::pAXX1His_6_ (P*_MXR1_*-Mxr1–His_6_ *hph*) P*_GAP_*::pPGBmh1Flag (P*_GAP_*-Bmh1–Flag *HIS4, KAN*) | This study |
| WT-PM | GS115 P*_PRM1_*::pPPP1HA (P*_PRM1_*-Prm1–HA *HIS4, KAN*) P*_GAP_*::pGGM1His_6_ (P*_GAP_*-Mit1–His_6_ *Sh ble*) | This study |
| Mxr1-YN/Mit1-YC | GS115 P*_GAP_*::pBIFC-Mxr1-YN (P*_GAP_*-Mxr1–YN *hph*) P*_GAP_*::pBIFC-Mit1-YC (P*_GAP_*-Mit1–YC *sh ble*) | This study |
| Mxr1-YN/YC-Mit1 | GS115 P*_GAP_*::pBIFC-Mxr1-YN (P*_GAP_*-Mxr1–YN *hph*) P*_GAP_*::pBIFC-YC-Mit1 (P*_GAP_*-YC–Mit1 *sh ble*) | This study |
| YN-Mxr1/Mit1-YC | GS115 P*_GAP_*::pBIFC-YN-Mxr1 (P*_GAP_*-YN–Mxr1 *hph*) P*_GAP_*::pBIFC-Mit1-YC (P*_GAP_*-Mit1–YC *sh ble*) | This study |
| YN-Mxr1/YC-Mit1 | GS115 P*_GAP_*::pBIFC-YN-Mxr1 (P*_GAP_*-YN–Mxr1 *hph*) P*_GAP_*::pBIFC-YC-Mit1 (P*_GAP_*-YC–Mit1 *sh ble*) | This study |
| Prm1-YN/Mit1-YC | GS115 P*_GAP_*::pBIFC-Prm1-YN (P*_GAP_*-Prm1–YN *hph*) P*_GAP_*::pBIFC-Mit1-YC (P*_GAP_*-Mit1–YC *sh ble*) | This study |
| Prm1-YN/YC-Mit1 | GS115 P*_GAP_*::pBIFC-Prm1-YN (P*_GAP_*-Prm1–YN *hph*) P*_GAP_*::pBIFC-YC-Mit1 (P*_GAP_*-YC–Mit1 *sh ble*) | This study |
| YN-Prm1/Mit1-YC | GS115 P*_GAP_*::pBIFC-YN-Prm1 (P*_GAP_*-YN–Prm1 *hph*) P*_GAP_*::pBIFC-Mit1-YC (P*_GAP_*-Mit1–YC *sh ble*) | This study |
| YN-Prm1/YC-Mit1 | GS115 P*_GAP_*::pBIFC-YN-Prm1 (P*_GAP_*-YN–Prm1 *hph*) P*_GAP_*::pBIFC-YC-Mit1 (P*_GAP_*-YC–Mit1 *sh ble*) | This study |
| Mxr1-YN/Prm1-YC | GS115 P*_GAP_*::pBIFC-Mxr1-YN (P*_GAP_*-Mxr1–YN *hph*) P*_GAP_*::pBIFC-Prm1-YC (P*_GAP_*-Prm1–YC *sh ble*) | This study |
| Mxr1-YN/YC-Prm1 | GS115 P*_GAP_*::pBIFC-Mxr1-YN (P*_GAP_*-Mxr1–YN *hph*) P*_GAP_*::pBIFC-YC-Prm1 (P*_GAP_*-YC–Prm1 *sh ble*) | This study |
| YN-Mxr1/Prm1-YC | GS115 P*_GAP_*::pBIFC-YN-Mxr1 (P*_GAP_*-YN–Mxr1 *hph*) P*_GAP_*::pBIFC-Prm1-YC (P*_GAP_*-Prm1–YC *sh ble*) | This study |
| YN-Mxr1/YC-Prm1 | GS115 P*_GAP_*::pBIFC-YN-Mxr1 (P*_GAP_*-YN–Mxr1 *hph*) P*_GAP_*::pBIFC-YC-Prm1 (P*_GAP_*-YC–Prm1 *sh ble*) | This study |
| Mxr1-YN/C4qzn3-YC | GS115 P*_GAP_*::pBIFC-Mxr1-YN (P*_GAP_*-Mxr1–YN *hph*) P*_GAP_*::pBIFC-C4qzn3-YC (P*_GAP_*-C4qzn3–YC *sh ble*) | This study |
| Mxr1-YN/YC-C4qzn3 | GS115 P*_GAP_*::pBIFC-Mxr1-YN (P*_GAP_*-Mxr1–YN *hph*) P*_GAP_*::pBIFC-YC-C4qzn3 (P*_GAP_*-YC–C4qzn3 *sh ble*) | This study |

**Table S2** Plasmids used in this study.

| Plasmid | Characteristic(s)^a^ | Source or reference |
| --- | --- | --- |
| pUC18 | Ampicillin^R^; *E. coli* subcloning vector | Invitrogen |
| pPIC3.5K | Ampicillin^R^, G418^R^; P*_AOX1_*-based expression vector | Invitrogen |
| pPIC6A | Blasticidin^R^; P*_AOX1_*-based expression vector | Invitrogen |
| pGAPZB | Zeocin^R^; P*_GAP_*-based expression vector | Invitrogen |
| pRDM054 | Ampicillin^R^, hygromycin^R^ | (1) |
| pAG32 | Ampicillin^R^, hygromycin^R^ | (2) |
| pMIT1-del | pPIC3.5K derivative containing the *MIT1* deletion cassette | This study |
| pPRM1-del | pUC18 derivative containing the *PRM1* deletion cassette | This study |
| pMXR1-del | pUC18 derivative containing the *MXR1* deletion cassette | This study |
| pGMM1 | pGAPZB derivative containing P*_MIT1_*-Mit1 expression cassette | This study |
| pAPP1 | pAG32 derivative containing P*_PRM1_*-Prm1 cassette | This study |
| pPXX1 | pPGG derivative containing P*_MXR1_*-Mxr1 cassette | This study |
| pGM-Mpp1 | pGAPZB derivative containing P*_MIT1_*-Mpp1 expression cassette | This study |
| pGGLMit1 | pGAPZB derivative containing P*_GAP_*-GFP–Mit1 expression cassette | This study |
| pGGLPrm1 | pGAPZB derivative containing P*_GAP_*-GFP–Prm1 expression cassette | This study |
| pP-GFP | pPIC3.5K derivative containing P*_AOX1_*-GFP expression cassette | (3) |
| pPGG | pP-GFP derivative containing P*_GAP_*-GFP expression cassette | This study |
| pGMG | pGAPZB derivative containing P*_MIT1_*-GFP expression cassette | This study |
| pAPG | pGAPZB derivative containing P*_PRM1_*-GFP expression cassette | This study |
| pPWBS | pPIC3.5K derivative containing P*_GCW14_*-BFP–SKL expression cassette | This study |
| pGMMΔZF | pGAPZB derivative containing P*_MIT1_*-Mit1ΔZF–Flag cassette | This study |
| pGMMΔRR1 | pGAPZB derivative containing P*_MIT1_*-Mit1ΔRR1–Flag cassette | This study |
| pGMMΔUR1 | pGAPZB derivative containing P*_MIT1_*-Mit1ΔUR1–Flag cassette | This study |
| pGMMΔRR2 | pGAPZB derivative containing P*_MIT1_*-Mit1ΔRR2–Flag cassette | This study |
| pGMMΔUR2 | pGAPZB derivative containing P*_MIT1_*-Mit1ΔUR2–Flag cassette | This study |
| pGMMΔUR3 | pGAPZB derivative containing P*_MIT1_*-Mit1ΔUR3–Flag cassette | This study |
| pGMMΔRR3 | pGAPZB derivative containing P*_MIT1_*-Mit1ΔRR3–Flag cassette | This study |
| pGMMΔUR4 | pGAPZB derivative containing P*_MIT1_*-Mit1ΔUR4–Flag cassette | This study |
| pGMMΔRR1ΔRR2 | pGAPZB derivative containing P*_MIT1_*-Mit1ΔRR1ΔRR2–Flag cassette | This study |
| pGMMΔRR1ΔRR3 | pGAPZB derivative containing P*_MIT1_*-Mit1ΔRR1ΔRR3–Flag cassette | This study |
| pGMMΔRR2ΔRR3 | pGAPZB derivative containing P*_MIT1_*-Mit1ΔRR2ΔRR3–Flag cassette | This study |
| pGMMΔRR1ΔRR2ΔRR3 | pGAPZB derivative containing P*_MIT1_*-Mit1ΔRR1ΔRR2ΔRR3–Flag cassette | This study |
| pP6GX1 | pPIC6A derivative containing P*_GAP_*-Mxr1–HA cassette | This study |
| pAGM1 | pAG32 derivative containing P*_GAP_*-Mit1–HA cassette | This study |
| pPGP1 | pPIC3.5K derivative containing P*_GAP_*-Prm1–HA cassette | This study |
| pGMM1Flag | pGAPZB derivative containing P*_MIT1_*-Mit1–Flag cassette | This study |
| pGGM1Flag | pGAPZB derivative containing P*_GAP_*-Mit1–Flag cassette | This study |
| pPPP1HA | pPIC3.5K derivative containing P*_PRM1_*-Prm1–HA cassette | This study |
| pGGM1His6 | pGAPZB derivative containing P*_GAP_*-Mit1–His_6_ cassette | This study |
| pAXX1His6 | pAG32 derivative containing P*_MXR1_*-Mxr1–His_6_ cassette | This study |
| pPGBmh1Flag | pPIC3.5K derivative containing P*_GAP_*-Bmh1–Flag cassette | This study |
| pUT18C | Ampicillin^R^, P*_LAC_* based expression vector with T18 domain of *Bordella* *pertussis* adenylate cyclase | Euromedex |
| pKT25 | Kanamycin^R^, P*_LAC_* based expression vector with T25 domain of *Bordella* *pertussis* adenylate cyclase | Euromedex |
| pUT18C-zip | pUT18C derivative containing P*_LAC_*-T18–zip expression cassette | Euromedex |
| pKT25-zip | pKT25 derivative containing P*_LAC_*-T25–zip expression cassette | Euromedex |
| pUT18C-Prm1 | pUT18C derivative containing P*_LAC_*-T18–Prm1 expression cassette | This study |
| pUT18C-Mxr1 | pUT18C derivative containing P*_LAC_*-T18–Mxr1 expression cassette | This study |
| pKT25-Mit1 | pKT25 derivative containing P*_LAC_*-T25–Mit1 expression cassette | This study |
| pKT25-Prm1 | pKT25 derivative containing P*_LAC_*-T25–Prm1 expression cassette | This study |
| pKT25-Mxr1 | pKT25 derivative containing P*_LAC_*-T25–Mxr1 expression cassette | This study |
| pKT25-C4qzn3 | pKT25 derivative containing P*_LAC_*-T25–C4qzn3 expression cassette | This study |
| pBIFC-Mit1–YC | pGAPZ B derivative containing P*_GAP_*-Mit1–YC expression cassette | This study |
| pBIFC-YC–Mit1 | pGAPZ B derivative containing P*_GAP_*-YC–Mit1 expression cassette | This study |
| pBIFC-Mxr1–YN | pAG32 derivative containing P*_GAP_*-Mxr1–YN expression cassette | This study |
| pBIFC-YN–Mxr1 | pAG32 derivative containing P*_GAP_*-YN–Mxr1 expression cassette | This study |
| pBIFC-Prm1–YN | pAG32 derivative containing P*_GAP_*-Prm1–YN expression cassette | This study |
| pBIFC-YN–Prm1 | pAG32 derivative containing P*_GAP_*-YN–Prm1 expression cassette | This study |
| pBIFC-Prm1–YC | pGAPZ B derivative containing P*_GAP_*-Prm1–YC expression cassette | This study |
| pBIFC-YC–Prm1 | pGAPZ B derivative containing P*_GAP_*-YC–Prm1 expression cassette | This study |
| pBIFC-C4qzn3–YC | pGAPZ B derivative containing P*_GAP_*-C4qzn3–YC expression cassette | This study |
| pBIFC-YC–C4qzn3 | pGAPZ B derivative containing P*_GAP_*-YC–C4qzn3 expression cassette | This study |

**^a^** R indicates resistance to indicated antimicrobial agent.

**Table S3** Oligonucleotide primers used in this study.

| Primer | Sequences (5’-3’)^b^ |
| --- | --- |
| *Primers used in the construction of Δmit1, Δprm1, and Δmxr1 strains* | |
| MIT1Up5 | CGGGATCCAATGAGGGTGCGGTGGTG |
| MIT1Up3 | GACAAGGCAAGCTGGCTGTTATATAGAATC |
| Hyg5 | ATATAACAGCCAGCTTGCCTTGTCCCCGCC |
| Hyg3 | TGATTGTGATTTGAGCTCGTTTTCGACACT |
| MIT1Do5 | CGGAATTCAAATCACAATCACCATTA |
| MIT1Do3 | ACGCGTCGACGCATGCCCTCTCCTTAGA |
| PRM1Up5 | CCGGAATTCTGTCCTCCTCTGCTGTTT |
| PRM1Up3 | CGCGGATCCTTTAGTTATAAAGAAGGGAGA |
| Zeo5 | CGCGGATCCCCCACACACCATAGCTTC |
| Zeo3 | ACGCGTCGACTTGGTCTCCAGCTTGCAA |
| PRM1Do5 | ACGCGTCGACAATGATTGGACCACTGCG |
| PRM1Do3 | ACATGCATGCATCCCAATGACACCACAA |
| MXR1Up5 | CCGGAATTCTAGGTTTCAGCGGTCTTTGG |
| MXR1Up3 | CGCGGATCCTGTGCGTGGGATAAAGTCAT |
| MXR1Do5 | ACGCGTCGACTTAATGAATTATGATTTTGTTTG |
| MXR1Do3 | ACATGCATGCATGGAGGTTCATTAGTTTGC |
| *Primers used in the construction of MIT1, PRM1, and MXR1 complementation strains* | |
| BlnI-PMIT1-5 | CGTCCCTAGGACAGAATCTGGAGGTGTAAAC |
| MIT1-3-SalI | ACGCGTCGACCTATTCTTCAACATTCCAGTAGT |
| SacI-PPRM1-5 | AACGAGCTCTCGTAATCCTTGAGTTTTTTCTCCG |
| PRM1-3-XhoI | CCGCTCGAGTTAACTGTCAAAATTTATTGTATC |
| PMXR1-5 | CGCCCGTTACCGTCCCTAGGAATGGCGGATGTCGAGTT |
| MXR1-3 | GAATTAATTCGCGGCCGCCCTAGGGACACCACCATCTAGTCGG |
| XhoI-AOX1TT-5 | CCGCTCGAGGTTTTAGCCTTAGACATG |
| AOX1TT-3-SpeI | GGACTAGTGCACAAACGAAGGTCTCA |
| *Primers used in the construction of Mpp1 complementation strains* | |
| BlnI-PMIT1-5 | CGTCCCTAGGACAGAATCTGGAGGTGTAAAC |
| PMIT1-o3-Mpp1 | TCGTCTCTGGAAATGGACATTGGCTGTTATATAGAATCTTTG |
| MPP1-o5-PMIT1 | CAAAGATTCTATATAACAGCCAATGTCCATTTCCAGAGACGA |
| MPP1-3-SalI | ACGCGTCGACTCACTTGTCGTCGTCGTCCTTGTAGTCAGAGCCGCCGCCACCGCACTCGCGTTTCCAGA |
| *Primers used in the construction of strains expressing GFP–Mit1 or GFP–Prm1 fusion protein* | |
| NotI-LMIT1-5 | AAGGAAAAAAGCGGCCGCTCTAGTACCGCAGCCCCAATCAAGGAAG |
| MIT1-3-SalI | ACGCGTCGACCTATTCTTCAACATTCCAGTAGTC |
| NotI-LPRM1-5 | AAGGAAAAAAGCGGCCGCTCTCCTCCTAAACATCGGCTGGAGCAG |
| PRM1-3-SalI | ACGCGTCGACCTAACTGTCAAAATTTATTGTATCTGGC |
| *Primers used in the construction of BFP–SKL expression strains* | |
| SacI-PGCW14-5 | CGAGCTCCACAATCAAGGTGAGTCCAG |
| PGCW14-3-EcoRI | CCGGAATTCTTTTGTTGTTGAGTGAAGCG |
| EcoRI-BFP-5 | CCGGAATTCACCATGGGTAGCAAGGGCGAGGAACTG |
| BFP-3-NotI | ATTTGCGGCCGCTTATAATTTGGACAGCTC |
| *Primers used in the construction of domain-deletion mutants: Flanking primers* | |
| KpnI-PMIT1-5 | CGGGGTACCACTCAAAGGACTGC |
| MIT1-3-SalI | ACGCGTCGACCTATTCTTCAACATTCCAGTAGT |
| *Primers used in the construction of domain-deletion mutants: Mutagenic primers* | |
| M-ZFo3 | AGAAATCCTTCATGGAGTTGTTAACTTTTTTCACAGGTGCAGCGT |
| M-ZFo5 | ACGCTGCACCTGTGAAAAAAGTTAACAACTCCATGAAGGATTTCT |
| M-RR1o3 | GCCAGAGTTTGAACCTATCAAGTCCTTGCCTAAGAAATCCTTCATGG |
| M-RR1o5 | CCATGAAGGATTTCTTAGGCAAGGACTTGATAGGTTCAAACTCTGGC |
| M-UR1o3 | GAATTTCCATGCTTTTTTTGAGATGAACCTATCAAGTCTTGGAAGT |
| M-UR1o5 | ACTTCCAAGACTTGATAGGTTCATCTCAAAAAAAGCATGGAAATTC |
| M-RR2o3 | GATGAGTTCGAAGACTGGTCCTTTTTTTGAGATGCTTCTTGAACT |
| M-RR2o5 | AGTTCAAGAAGCATCTCAAAAAAAGGACCAGTCTTCGAACTCATC |
| M-UR2o3 | GCTTCACATCTTGAATGGTCTTGTATGAGTTCGAAGACTGGTCCTTAG |
| M-UR2o5 | CTAAGGACCAGTCTTCGAACTCATACAAGACCATTCAAGATGTGAAGC |
| M-UR3o3 | ATTGAAGCAGAAACTGTAGTAAAGGGCTTCACATCTTGAATGGTCTTGTAG |
| M-UR3o5 | CTACAAGACCATTCAAGATGTGAAGCCCTTTACTACAGTTTCTGCTTCAAT |
| M-RR3o3 | ACAAATGATCACCAGAAGCGTGGTGAGTAAAGGGGATTTTGTATAATCGT |
| M-RR3o5 | ACGATTATACAAAATCCCCTTTACTCACCACGCTTCTGGTGATCATTTGT |
| M-UR4-SalI | ACGCGTCGACCTAACCAGAAGCGTGGTGGTCTG |
| *Primers used in the construction of strains in ChIP assay* | |
| KpnI-PMIT1-5 | CGGGGTACCACTCAAAGGACTGC |
| MIT1-3-Flag-SalI | ACGCGTCGACCTACTTGTCGTCGTCGTCCTTGTAGTCAGAGCCGCCGCTTCTTCAACATTCCAGTAGT |
| AsuII-PPRM1-5 | TTCCTTCGAATTCCTTCGAAGAATTTTGTTAC |
| PRM1-3-HA-XhoI | CCGCTCGAGCTAAGCGTAGTCTGGAACGTCGTATGGGTAAGAGCCGCCGCCACCACTGTCAAAATTTATTGTATC |
| *Primers used in the construction of strains in Ni^2+^-NTA pulldown assay* | |
| EcoRI-MIT1-5 | CCGGAATTCACCATGGGTAGTACCGCAGCCCCAAT |
| MIT1-3His-XhoI | CCGCTCGAGCTTCTTCAACATTCCAGTAGTC |
| PMXR1Fhyg | CCATCCAGTGTCGAAAACGAGCTCAGATCTTTTTTGTAATGGCGG |
| MXR1Rhyg | AGCTGGCGGCCGCCGCGGCTCGAGCTAATGATGATGATGATGATGGAC |
| BamHI-Bmh1-5 | CGCGGATCCACCATGGGTTCAAGAGAAGATTCTGTTTAT |
| Bmh1-Flag3-NotI | ATTTGCGGCCGCCTACTTGTCGTCGTCGTCCTTGTAGTCAGAGCCGCCGCCACCCTCTTCATCTTTGGGAGCAG |
| *Primers used in qPCR assay* | |
| RT-ACT1F | CTCCAATGAACCCAAAGTCCAAC |
| RT-ACT1R | GACAAAACGGCCTGAATAGAAAC |
| RT-AOX1F | GAGGCCAGAGCCTTGGAA |
| RT-AOX1R | CCTTCGTTCTTTGCAGTT |
| RT-AOX2F | GAGGCCAGAGCTTACGAG |
| RT-AOX2R | CCTTCGTTTCTTCCAGCA |
| RT-DAS1F | GGTGACGAGTTAGTAAAG |
| RT-DAS1R | CCTCTAACACGAGAAAGG |
| RT-DAS2F | GGTGATCAACTAGTTGCT |
| RT-DAS2R | CCTCTAATACGGGCCTTT |
| RT-CATF | GCTAATCACGCTAACAAT |
| RT-CATR | GGCAAGAGAATCAATCAA |
| RT-FLDF | TTGGTTCAGGACTATCTT |
| RT-FLDR | GTAATCACAGCACGAATA |
| RT-FDHF | TTCCACTCCATTCCATCC |
| RT-FDHR | CAACGACCAACAACTTCA |
| RT-PEX3F | ATCCTTGCTGATTGTATT |
| RT-PEX3R | CACCATTAGACTCTTGAT |
| RT-PEX5F | GGCTCGCTATAATCTTGG |
| RT-PEX5R | CAACACCTTCAACCTCAT |
| RT-PEX14F | GAGGTTAAGGAGGCATTG |
| RT-PEX14R | AGAATACGATGACACTTGG |
| RT-PMP20F | TTCCACATTCCATCATCT |
| RT-PMP20R | GCAACGACAATAAATCTCT |
| RT-PMP47F | AACAACCTACCAACACTAT |
| RT-PMP47R | GCTGATACCAACTCTTCT |
| RT-PEX10F | GGCAGTTCTATAACATATCC |
| RT-PEX10R | CCTCCTAGTAATTCGTAGT |
| RT-MIT1F | GACTAATGACGATGAACTAAG |
| RT-MIT1R | TGCTGTTGTTGGTAGAAT |
| RT-PRM1F | CGAACTTGATGATGAGAACA |
| RT-PRM1R | CATTGGCTATTCCTGAACTG |
| RT-MXR1F | ATGCTGCTGATGCTATGA |
| RT-MXR1R | GCGGTCTGAATCGTTATTAC |
| *Primers used in the amplification of fragments for EMSAs and DNase I footprinting assays* | |
| PAOX1-W1F | Cy5-AGATCTAACATCCAAAGACG |
| PAOX1-W1R | TAGCCTAATAGAAGGAATTG |
| PAOX1-W2F | Cy5-CAATTCCTTCTATTAGGCTA |
| PAOX1-W2R | CAAGACAGCGTTTAAACTGT |
| PAOX1-W3F | Cy5-ACAGTTTAAACGCTGTCTTG |
| PAOX1-W3R | ATTTTTGAGCATTCGTCAAT |
| PAOX1-W4F | Cy5-ATTGACGAATGCTCAAAAAT |
| PAOX1-W4R | TTGCTGTCAAGTAGGGGTTA |
| PMIT1-AF | Cy5-ACAGAATCTGGAGGTGTA |
| PMIT1-AR | ATTGGGAGTGTTCAAGTG |
| PMIT1-BF | Cy5-TAGCTGGCATCAATGCCA |
| PMIT1-BR | CTACGTTCATTCCCTTTC |
| PMIT1-CF | Cy5-GAGGGTGCGGTGGTGGGG |
| PMIT1-CR | GCTAAACAACATCTGCTT |
| PMIT1-DF | Cy5-GTTCTTCCCGTTCCGCTC |
| PMIT1-DR | GTACCAAATGACCAATGG |
| PMIT1-EF | Cy5-GCGATCGCCCCAACCCCT |
| PMIT1-ER | TGGCTGTTATATAGAATC |
| PPRM1-AF | Cy5-GATAGCACTCTGGATTGAT |
| PPRM1-AR | TCTTCGCGAGCATATTCCATCAG |
| PPRM1-BF | Cy5-CTGATGGAATATGCTCGCGAAGA |
| PPRM1-BR | CGCGACTATGACAGCCATTTAAGAT |
| PPRM1-CF | Cy5-ATCTTAAATGGCTGTCATAGTCGCG |
| PPRM1-CR | CACCAGCACAAGGGATGAAGTGC |
| M13F-47 | FAM-CGCCAGGGTTTTCCCAGTCACGAC |
| M13R-48 | GAGCGGATAACAATTTCACACAGG |
| *Primers used in the ChIP-qPCR assay* | |
| PACT1-F | GTGACCTACCGATAAGTTC |
| PACT1-R | TTACTGGACGCTCTACAA |
| A1-F | AGATCTAACATCCAAAGACG |
| A1-R | ACGGTCTGCTGCTAGTGTAT |
| A2-F | ATACACTAGCAGCAGACCGT |
| A2-R | TTGGAATGAGCGAGCTCCAA |
| A3-F | TTGGAGCTCGCTCATTCCAA |
| A3-R | GAGCTTGTTGCATTCGGAAA |
| A4-F | TTTCCGAATGCAACAAGCTC |
| A4-R | AGCGTTTAAACTGTCAGTTT |
| A5-F | AAACTGACAGTTTAAACGCT |
| A5-R | TGGAAGTTTCTTTTTGACCA |
| A6-F | TGGTCAAAAAGAAACTTCCA |
| A6-R | GGGGTTCAGAAGCGATAGAG |
| A7-F | CTCTATCGCTTCTGAACCCC |
| A7-R | AGTATTCCCACCAGAATCTT |
| A8-F | AAGATTCTGGTGGGAATACT |
| A8-R | GGTTTAAGACAGGGCAGCTT |
| A9-F | AAGCTGCCCTGTCTTAAACC |
| A9-R | CGTTTCGAATAATTAGTTG |
| *Primers used in the construction of strains in B2H assay* | |
| pUT18C5R | GGGATCCTCTAGAGTCGACCTGCAG |
| pUT18C3F | CGGGTACCGAGCTCGAATTCATCG |
| Prm1Fb18 | GGTCGACTCTAGAGGATCCCATGCCTCCTAAACATCGGCTGGAGC |
| Prm1Rb18 | GAATTCGAGCTCGGTACCCGTTAACTGTCAAAATTTATTGTATC |
| Mxr1Fb18 | GGTCGACTCTAGAGGATCCCATGAGCAATCTACCCCCAAC |
| Mxr1Rb18 | GAATTCGAGCTCGGTACCCGCTAGACACCACCATCTAGTCGG |
| pKT25-5R | GGGATCCTCTAGAGTCGACCCTGC |
| pKT25-3F | CGGGTACCTAAGTAACTAAGAATTC |
| Mit1Fb25 | GGTCGACTCTAGAGGATCCCATGAGTACCGCAGCCCCAATC |
| Mit1Rb25 | CTTAGTTACTTAGGTACCCGCTATTCTTCAACATTCCAGTAG |
| Prm1Fb25 | GGTCGACTCTAGAGGATCCCATGCCTCCTAAACATCGGCTGGAGC |
| Prm1Rb25 | CTTAGTTACTTAGGTACCCGTTAACTGTCAAAATTTATTGTATC |
| Mxr1Fb25 | GGTCGACTCTAGAGGATCCCATGAGCAATCTACCCCCAAC |
| Mxr1Rb25 | CTTAGTTACTTAGGTACCCGCTAGACACCACCATCTAGTCGG |
| C4qzn3Fb25 | GGTCGACTCTAGAGGATCCCATGTCAAGAGAAGATTCTGTTTAT |
| C4qzn3Rb25 | CTTAGTTACTTAGGTACCCGTTACTCTTCATCTTTGGGAGCAG |
| pKT25-5F | GGCAATGCCGCCGGTATTCCA |
| pKT25-3R | CCTGTCCCTCCTGTTCAGCTA |
| pUT18C-5F | ACGGCGTGGCGGGGAAAAG |
| pUT18C-3R | GCGTTTCGGTGATGACGGTG |
| *Primers used in the construction of strains in BiFC assay* | |
| YFPC3-pGAPZ-5 | TACAAGTAGCTCGAGCCGCGGCGGCCG |
| pGAPZ-3-MIT1 | GGTACTCATACCCATGGTGAATTCCTCGT |
| pGAPZ-MIT1-5 | ACCATGGGTATGAGTACCGCAGCCCCAAT |
| MIT1-3-YFPC | GCACGCCGGACGTTCTTCAACATTCCAGTAGTC |
| MIT1-YFPC-5-1 | ACCTGAAACAGAAAGTCATGAACCACGACAAGCAGAAGAACGGCATC |
| MIT1-YFPC-5-2 | GAAGAACGTCCGGCGTGCAAAATCCCGAACGACCTGAAACAGAAAGTCA |
| YFPC-3- pGAPZ | CGCGGCTCGAGCTACTTGTACAGCTCGTCCATGC |
| MIT1-pGAPZ-5 | GAAGAATAGCTCGAGCCGCGGCGGCCG |
| pGAPZ-3-YFPC | CTTGTCCATACCCATGGTGAATTCCTCGT |
| pGAPZ-YFPC-5 | ACCATGGGTATGGACAAGCAGAAGAACGGCATC |
| YFPC-3-MIT1 | CATAGTACCACCAGAACCCTTGTACAGCTCGTCCATGC |
| YFPC-MIT1-5 | GGTTCTGGTGGTACTATGAGTACCGCAGCCCCAAT |
| MIT1-3-pGAPZ | CGGCTCGAGCTATTCTTCAACATTCCAGTAGTC |
| YFPN3-pAGM-5 | ATCGAGTAGCTCGAGCCGCGGCGGCCG |
| pAGM-3-MXR1 | AGTGGTCATACCCATGGTGAATTCCTCGT |
| pAGM-MXR1-5 | GAATTCACCATGGGTATGAGCAATCTACCCCCAACT |
| MXR1-3-YFPN | CATGGTGGCGATGGATCTGACACCACCATCTAGTCGGTT |
| MXR1-YFPN-5 | GTCAGATCCATCGCCACCATGGTGAGCAAGGGCGAGGAG |
| YFPN-3-pAGM | CGGCTCGAGCTACTCGATGTTGTGGCGGATC |
| YFPNF | AGATCCATCGCCACCATGGTGAGC |
| pAGMR | CATACCCATGGTGAATTCCTCGT |
| pAGM-PRM1-5 | GAATTCACCATGGGTATGCCTCCTAAACATCGGCTG |
| PRM1-3-YFPN | CATGGTGGCGATGGATCTACTGTCAAAATTTATTGTATCTG |
| MXR1-pAGM-5 | GGTGTCTAGCTCGAGCCGCGGCGGCCG |
| pAGM-3-YFPN | GCTCACCATACCCATGGTGAATTCCTCGT |
| pAGM-YFPN-5 | ACCATGGGTATGGTGAGCAAGGGCGAGGAG |
| YFPN-MXR1-5 | GGTTCTGGTGGTACTATGAGCAATCTACCCCCAACT |
| MXR1-3-pAGM | CGGCCGCCGCGGCTCGAGCTAGACACCACCATCTAGTCGGTT |
| pAGMF | TAGCTCGAGCCGCGGCGG |
| YFPNR | CATAGTACCACCAGAACC |
| YFPN-PRM1-5 | GGTTCTGGTGGTACTATGCCTCCTAAACATCGGCTG |
| PRM1-3-pAGM | CCGCCGCGGCTCGAGCTAACTGTCAAAATTTATTGTATCTG |
| YFPCF | CGTCCGGCGTGCAAAATCCCG |
| pGAPZR | CATACCCATGGTGAATTCCTCGT |
| pGAPZ-PRM1-5 | GAATTCACCATGGGTATGCCTCCTAAACATCGGCTG |
| PRM1-3-YFPC | GGATTTTGCACGCCGGACGACTGTCAAAATTTATTGTATCTG |
| pGAPZF | TAGCTCGAGCCGCGGCGGCCGC |
| YFPCR | CATAGTACCACCAGAACC |
| YFPC-PRM1-5 | GGTTCTGGTGGTACTATGCCTCCTAAACATCGGCTG |
| PRM1-3-pGAPZ | CCGCCGCGGCTCGAGCTAACTGTCAAAATTTATTGTATCTG |
| pGAPZ-C4qzn3-5 | GAATTCACCATGGGTATGTCAAGAGAAGATTCTGTTTAT |
| C4qzn3-3-YFPC | GGATTTTGCACGCCGGACGCTCTTCATCTTTGGGAGCAG |
| YFPC-C4qzn3-5 | GGTTCTGGTGGTACTATGTCAAGAGAAGATTCTGTTTAT |
| C4qzn3-3-pGAPZ | CCGCCGCGGCTCGAGCTACTCTTCATCTTTGGGAGCAG |

**^b^** The underlined nucleotide sequences are for restriction enzyme recognition sites.

**References**

1. Wang, Y., Xuan, Y., Zhang, P., Jiang, X., Ni, Z., Tong, L., Zhou, X., Lin, L., Ding, J., and Zhang, Y. (2009) Targeting expression of the catalytic domain of the kinase insert domain receptor (KDR) in the peroxisomes of *Pichia* *pastoris*. *FEMS Yeast Res.* **9**, 732-741

2. Goldstein, A. L., and McCusker, J. H. (1999) Three new dominant drug resistance cassettes for gene disruption in *Saccharomyces* *cerevisiae*. *Yeast* **15**, 1541-1553

3. Xuan, Y., Zhou, X., Zhang, W., Zhang, X., Song, Z., and Zhang, Y. (2009) An upstream activation sequence controls the expression of *AOX1* gene in *Pichia* *pastoris*. *FEMS Yeast Res.* **9**, 1271-1282
